# Octopamine neuron dependent aggression requires dVGLUT from dual-transmitting neurons

**DOI:** 10.1371/journal.pgen.1008609

**Published:** 2020-02-25

**Authors:** Lewis M. Sherer, Elizabeth Catudio Garrett, Hannah R. Morgan, Edmond D. Brewer, Lucy A. Sirrs, Harold K. Shearin, Jessica L. Williams, Brian D. McCabe, R. Steven Stowers, Sarah J. Certel

**Affiliations:** 1 Cellular, Molecular and Microbial Biology Graduate Program, University of Montana, Missoula, Montana, United States of America; 2 Division of Biological Sciences, Center for Structural and Functional Neuroscience, University of Montana, Missoula, Montana, United States of America; 3 Cell Biology and Neuroscience Department, Montana State University, Bozeman, Montana, United States of America; 4 Brain Mind Institute, Swiss Federal Institute of Technology (EPFL), Lausanne, Switzerland; National Centre for Biological Sciences, TIFR, INDIA

## Abstract

Neuromodulators such as monoamines are often expressed in neurons that also release at least one fast-acting neurotransmitter. The release of a combination of transmitters provides both “classical” and “modulatory” signals that could produce diverse and/or complementary effects in associated circuits. Here, we establish that the majority of *Drosophila* octopamine (OA) neurons are also glutamatergic and identify the individual contributions of each neurotransmitter on sex-specific behaviors. Males without OA display low levels of aggression and high levels of inter-male courtship. Males deficient for dVGLUT solely in OA-glutamate neurons (OGNs) also exhibit a reduction in aggression, but without a concurrent increase in inter-male courtship. Within OGNs, a portion of VMAT and dVGLUT puncta differ in localization suggesting spatial differences in OA signaling. Our findings establish a previously undetermined role for dVGLUT in OA neurons and suggests that glutamate uncouples aggression from OA-dependent courtship-related behavior. These results indicate that dual neurotransmission can increase the efficacy of individual neurotransmitters while maintaining unique functions within a multi-functional social behavior neuronal network.

## Author summary

Neurons communicate with each other via electrical events and the release of chemical signals. An emerging challenge in understanding neuron communication is the realization that many neurons release more than one type of chemical signal or neurotransmitter. Here we ask how does the release of more than one neurotransmitter from a single neuron impact circuits that control behavior? We determined the monoamine octopamine and the classical transmitter glutamate are co-expressed in the *Drosophila* adult CNS. By manipulating the release of glutamate in OA-glutamate neurons, we demonstrated glutamate has both separable actions and complementary actions with OA on aggression and reproductive behaviors respectively. Aggression is a behavior that is highly conserved between organisms and present in many human disease states, including depression and Alzheimer’s disease. Our results show that aggressive behavior requires the release of both neurotransmitters in dual-transmitting neurons and suggests within this set of neurons, glutamate may provide a new therapeutic target to modulate aggression in pathological conditions.

## Introduction

The classical view of information transfer for many decades was that each neuron released a single neurotransmitter, leading to the ‘one neuron, one transmitter’ hypothesis [1], formalized by John Eccles as Dale’s Principle [2]. Dale himself, however, recognized the possibility that neurons can release more than one molecule [3] and indeed, research from multiple systems and neuronal populations have established that many if not most, neurons release more than one neurotransmitter [4–7]. Dual neurotransmission has the potential to transform the way we consider the computation and transmission of information by neurons, circuits and networks. Presynaptically, the release of two neurotransmitters could impact information transfer by several mechanisms that are not mutually exclusive including; attenuating signals by modulating presynaptic autoreceptors, transmitting spatially distinct signals by segregating specific vesicle populations to different axon terminals, or conveying similar information through the release of both neurotransmitters from the same synaptic vesicle [8–11]. In addition, one vesicular neurotransmitter transporter can increase the packaging of the other neurotransmitter into the same synaptic vesicle (SV), a process called vesicular synergy [4, 12, 13]. At post-synaptic targets, the release of two transmitters can enhance the strength of the same signal and/or convey unique signals through spatially-restricted receptor expression and second messenger cascades [7, 14]. While recent studies have provided insight into these phenomena at the cellular level [11, 12, 15, 16], the behavioral relevance of co-transmission in normal as well as pathological conditions is an area of considerable complexity and interest.

The genetic tools of *Drosophila* provide the ability to genetically dissect the signaling properties of dual transmission on behavioral networks in general and upon the circuits that control aggression in particular. Aggression is a hardwired behavior that has evolved in the framework of defending or obtaining resources [17, 18]. Monoamines such as serotonin (5-HT), dopamine (DA), norepinephrine (NE) and octopamine (OA), the invertebrate homologue of NE, have powerful modulatory effects on aggression in systems ranging from insects and crustaceans to humans [19–23]. In humans, aggressive behavior can be expressed at extreme levels and out of context due to medical, neurologic and or psychiatric disorders including depression and schizophrenia [24–26]. Pharmacological agents that selectively manipulate monoamine signaling are used to treat anxiety and depression, yet these drugs are often ineffective, and in the case of serotonin/norepinephrine reuptake inhibitors (SNRIs) can induce side effects including increased aggression and impulsivity [25, 27–29].

At least two difficulties arise in targeting monoamines to achieve successful outcomes. First, monoamines can be released from synaptic vesicles (SVs) into the presynaptic cleft and by extrasynaptic release from large dense core vesicles (LDCVs) [30–33]. Thus, monoamines are recognized both as neurotransmitters and as neuromodulators that signal via diffusion [34, 35]. The second difficulty is that their effects are likely exerted through interactions with neuropeptides (neuropeptide Y and oxytocin are two examples) and with neurotransmitters including GABA and glutamate [5, 14, 36, 37]. Due in part to recent studies suggesting the expression of vesicular glutamate transporters (VGLUTs) can be altered by psychiatric medications [38–41] and the importance of dopamine neuron glutamate co-transmission on the schizophrenia resilience phenotype in mice [42], we generated new tools to identify and manipulate glutamate function in monoamine-expressing neurons.

We found that the majority of OA neurons within the *Drosophila* nervous system also express the vesicular neurotransmitter transporter for glutamate (dVGlut). Functionally, glutamate (GLU) co-expression could convey the same information by promoting the synaptic vesicle packing of OA or GLU may convey distinct information that is separate from the function of OA. In *Drosophila*, OA synthesis and release are essential for conserved social behaviors; males without OA display low levels of aggression and high levels of inter-male courtship [43–47]. We demonstrate that males deficient for dVGLUT solely in OA-glutamate neurons (OGNs) also exhibit a reduction in aggression, but without a concurrent increase in inter-male courtship. These results indicate both OA and dVGLUT are required in dual-transmitting neurons to promote aggression. However, only OA is required for the suppression of inter-male courtship and thus the function of dVGLUT in OGNs is not limited to vesicular synergy.

To ask if the separable effects of OA on courtship circuitry may be attributable to spatially distinct OA signals, we conditionally expressed a new epitope-tagged version of the *Drosophila* vesicular neurotransmitter transporter for monoamines (V5-tagged VMAT) in OGNs. While the majority of V5-VMAT and dVGLUT expression colocalize, VMAT is detected in distinct puncta without dVGLUT suggesting the possibility of separable signal transmission. Together, these results demonstrate the complex behavior of aggression requires both dVGLUT and OA in dual-transmitting neurons and suggests within monoamine neurons, GLU may provide a therapeutic target to modulate aggression in pathological conditions.

## Results

### dVGLUT is co-expressed in OA neurons

The co-expression of vesicular neurotransmitter transporters has been primarily used to identify dual-transmitting neurons[48–52]. To examine glutamatergic transmitter expression, we generated a monoclonal dVGLUT antibody and validated its specificity using a new *dVGlut* allele, *dVGlut*^*SS1*^. In homozygous *dVGlut*^*SS1*^ progeny, dVGLUT protein is not detectable (S1 Fig, Methods), thus demonstrating the specificity of the dVGLUT antibody. As dVGLUT expression is widespread and mainly found in synaptic terminals (S1 Fig), we used the Gal4-UAS system to identify monoamine neurons that express GLU. In this study, we focused specifically on OA neurons that co-express dVGLUT (OA-glutamate neurons (OGNs)).

**Fig 1:**
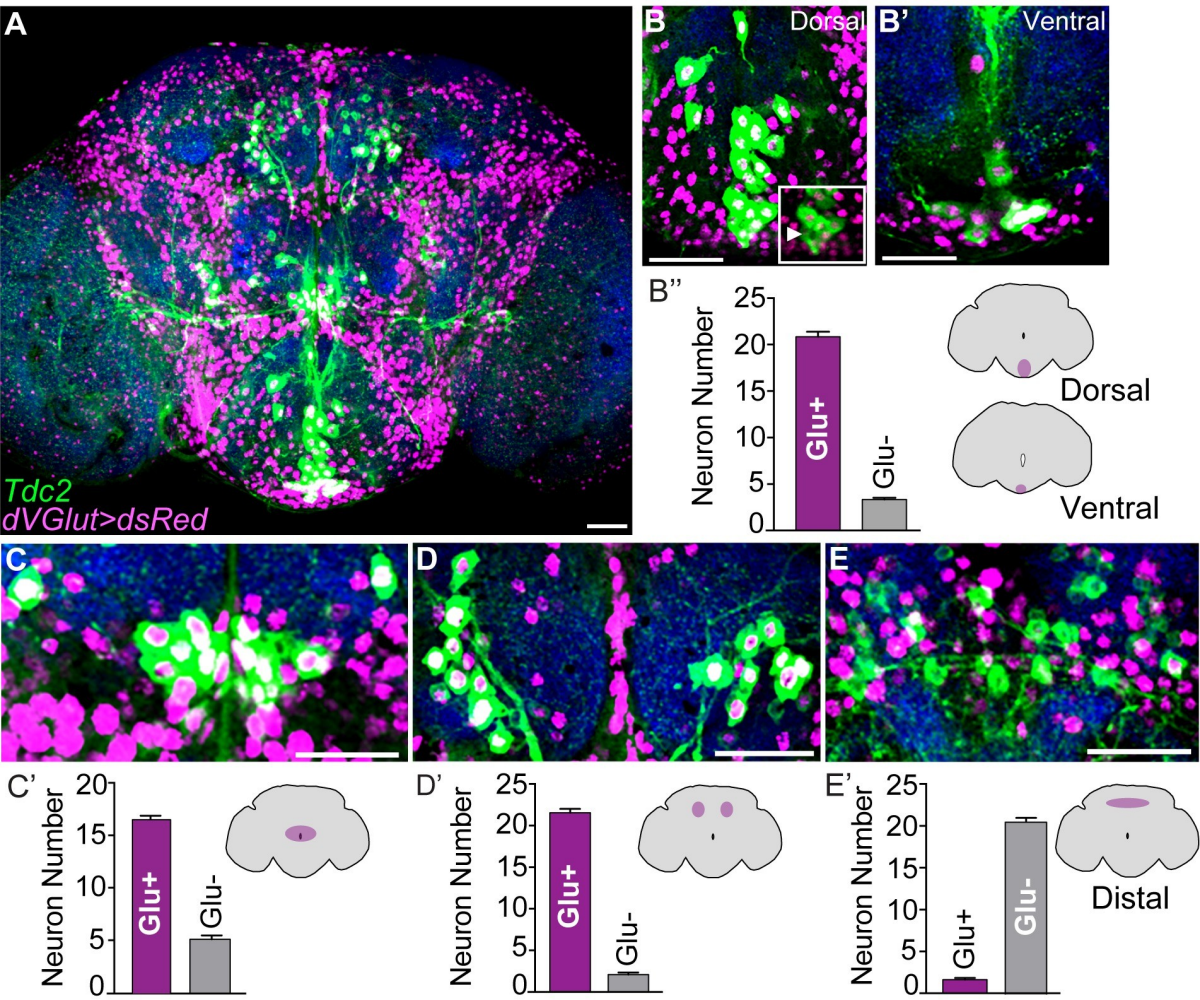
OA neurons co-express glutamate. **(A)** OA-glutamate co-expression in a *dVGlut>dsRed* male brain labeled with anti-Tdc2 (green). Anti-brp (nc82, blue) labels the neuropil. Scale bar = 10 μm. **(B-B’)** Dorsal (B) and ventral (B’) confocal sections of neurons co-expressing OA and dVGlut in the SEZ. Non-dVGlut positive neurons are indicated (B inset, arrowhead). **(B”)** Quantification of OGN SEZ co-expression. **(C-C’)** OGNs in the PENP and quantification. **(D-D’)** *dVGlut>dsRed* neurons expressing Tdc2 in the ASMP and quantification. **(E-E’)** Neurons co-expressing OA and glutamate in the PSMP and quantification. Scale bar = 20 μm for panels B-E.

Cell bodies of OGNs were visualized by a *UAS-dsRed*.*NLS* reporter under control of *dVGlut-gal4* (hereafter referred to as *dVGlut>dsRed*). OGNs were identified by antibodies to tyrosine decarboxylase 2 (TDC2) and tyramine β-hydroxylase (TβH) as OA is synthesized from the amino acid tyrosine via the action of Tdc and Tβh in invertebrates [46]. OGNs from 10 *dVGlut>dsRed* Tdc2-labeled male brains were quantified by the multi-point ImageJ tool followed by manual verification of each optical section. Within the brain, OA neurons that co-express glutamate are found in the subesophageal zone (SEZ), the periesophageal neuropils (PENP), the anterior (ASMP) and posterior superior medial protocerebrum (PSMP), and the protocerebral bridge (Fig 1A–1E, S1 Table). Co-expression occurs in each region of interest (Fig 1A–1E). Tβh and *dVGlut>dsRed* co-localization (S2 Fig) provides further support that glutamate is found in OA-expressing neurons.

In the adult ventral nervous system (VNS), the thoracic Tdc2+ neurons that innervate skeletal muscles express glutamate (S3 Fig). In the abdominal ganglia, all but 2–3 Tdc2+ neurons express dVGlut (S3 Fig) consistent with the previous finding of OA-glutamate co-expression in abdominal neurons [53]. After detecting no reporter expression from a *Tβh-gal4* driver, dVGLUT cell body expression in OGNs was detected in brains from *tdc2-gal4;UAS-dsRed* adults (S4 Fig). In total, this analysis reveals that of the ~100 OA neurons in the *Drosophila* adult nervous system, about 70% express dVGLUT.

### dVGLUT is not required for OA neuron identity

**Fig 2:**
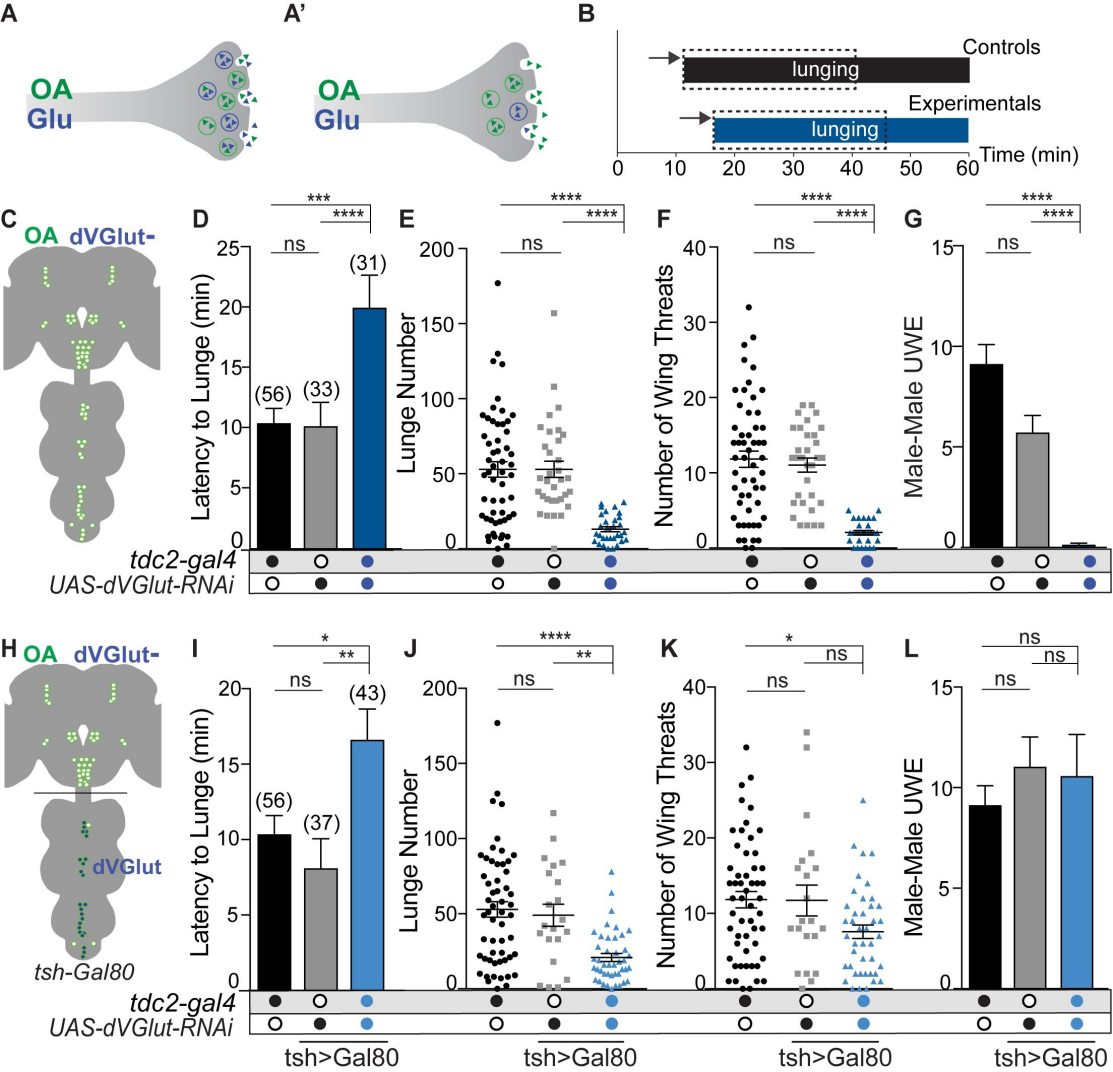
Male aggression requires dVGLUT function in OGNs. **(A)** dVGLUT reduction in OGNs through RNAi. **(B)** Behaviors for control and experimental male pairs were scored for thirty minutes beginning with the first lunge. **(C)** Schematic illustrating the brain and VNS OGNs**. (D)** Latency to lunge increased in *tdc2>dVGlut-RNAi* males (all statistical tests are Kruskal-Wallis with Dunn’s multiple comparisons test, (*p<0.05, **p<0.01, ***p<0.001, ****p<0.0001). **(E)** *tdc2>dVGlut-RNAi* males displayed a decrease in the average number of lunges. **(F)** Wing threats were reduced in *tdc2-dVGlut-RNAi* males. **(G)** *tdc2-dVGlut-RNAi* males did not exhibit inter-male courtship (unilateral wing extensions = UWE). **(H)** Schematic illustrating the addition of *tsh>Gal80* limits dVGLUT reduction to brain OGNs. **(I)** Latency to lunge by *tdc2-gal4/tsh>Gal80;UAS-dVGlut-RNAi* males is significantly longer than controls. **(J)** Lunge number by *tdc2-gal4/tsh>Gal80;UAS-dVGlut-RNAi* males decreases as compared to controls. **(K)** Wing threat number was rescued to *UAS-dVGlut-RNAi* control levels. **(L)** Male-male UWE was rescued to control levels. N values for each genotype, panels D, I. Error bars denote s.e.m.

To reduce glutamate function solely in OGNs, a UAS-driven inverted repeat transgene targeting *dVGlut* (*UAS-dVGlut-RNAi)* was expressed under control of the *tdc2-gal4* driver (hereafter *tdc2>dVGlut-RNAi*) (Fig 2A and 2B). The effectiveness of this *UAS-dVGlut-RNAi* line has been verified at the transcript level through RT-qPCR ([12] and S5 Fig) and functionally as the frequency of miniature excitatory postsynaptic potentials (mEPSP) were reduced by this dVGlut RNAi in presynaptic glutamatergic larval motor neurons [12]. As the loss of VGLUT2 in vertebrate dopamine-glutamate dual transmitting neurons impairs survival and differentiation *in vitro* [49, 54], we examined OGNs in *tdc2>dsRed*>*dVGlut-RNAi* adults and did not observe obvious changes in OGN survival nor distribution (S5 Fig). In addition, OGN neurotransmitter differentiation was retained as *tdc2>dVGlut-RNAi>dsRed* neurons express Tdc2 (S5 Fig). Neurons labeled by this *tdc2-gal4* whether in the brain or VNS are all Tdc2+ (S6A and S6B Fig).

### Reducing glutamate in OGNs decreases male aggression and inter-male courtship

We and others previously demonstrated OA is required for two distinct social male behaviors; the promotion of aggression, and the inhibition of intermale courtship [43, 46, 55, 56]. To address whether dVGLUT performs a related or separable role in these OA-dependent behaviors, we quantified changes in aggression and intermale courtship. Fights between pairs of *tdc2>dVGlut-RNAi* males, and transgenic controls were recorded and multiple agonistic parameters quantified including: latency to the first lunge, number of lunges, and number of agonistic wing threats (Fig 2A, [57, 58]). As behavioral patterns are scored for 30 minutes after the first lunge, each male pair has the same amount of time to exhibit aggressive events or inter-male courtship (Fig 2B).

Males with decreased dVGLUT in OGNs neurons exhibited a significant reduction in aggression as measured by lower numbers of lunges and wing threats, and an increase in the latency to initiate aggression (Fig 2D–2F). These aggression deficits are the same as in males that lack OA [43, 46, 47]. Importantly, the locomotor activity of *tdc2>dVGlut-RNAi* adults during the aggression assay did not differ from *dVGlut-RNAi* controls (S7A Fig).

Interactions between control male pairings within a fight can include low levels of intermale courtship as measured by unilateral wing extensions (UWE, the courtship song motor pattern). Males without OA exhibit high levels of inter-male courtship[43, 55, 56] and previously, we determined the function of three OA-FruM+ neurons is required to suppress intermale courtship [55]. If dVGLUT is only needed to enhance monoamine vesicular packaging and thus modulate OA function, we would expect males with reduced *dVGlut* levels to display the same behavioral deficits, i.e. high levels of inter-male courtship. However, *tdc2>dVGlut-RNAi* males did not exhibit inter-male courtship (Fig 2G). These results suggest; 1) dVGLUT is required in OGNs to promote aggression, and 2) dVGLUT is not required to suppress inter-male courtship.

### Aggression requires dVGLUT function in OA-GLU brain neurons

In the adult, motor neurons innervating leg and wing muscles express glutamate [59]. Therefore, the observed behavioral deficits in *tdc2>dVGlut-RNAi* males may reflect impairments at the neuromuscular junction. To address this possibility, we spatially restricted expression of the dVGlut-RNAi transgene to the brain using the *teashirt-lexA 8xlexAop2-IVS-Gal80* (hereafter *tsh>Gal80)* transgenic combination (Fig 2H). The *tsh>Gal80* transgenic combination was effective at blocking Gal4-mediated transcription in the entire VNS including in OGNs that innervate muscles required for courtship and wing threat behaviors (S8 Fig).

**Fig 3:**
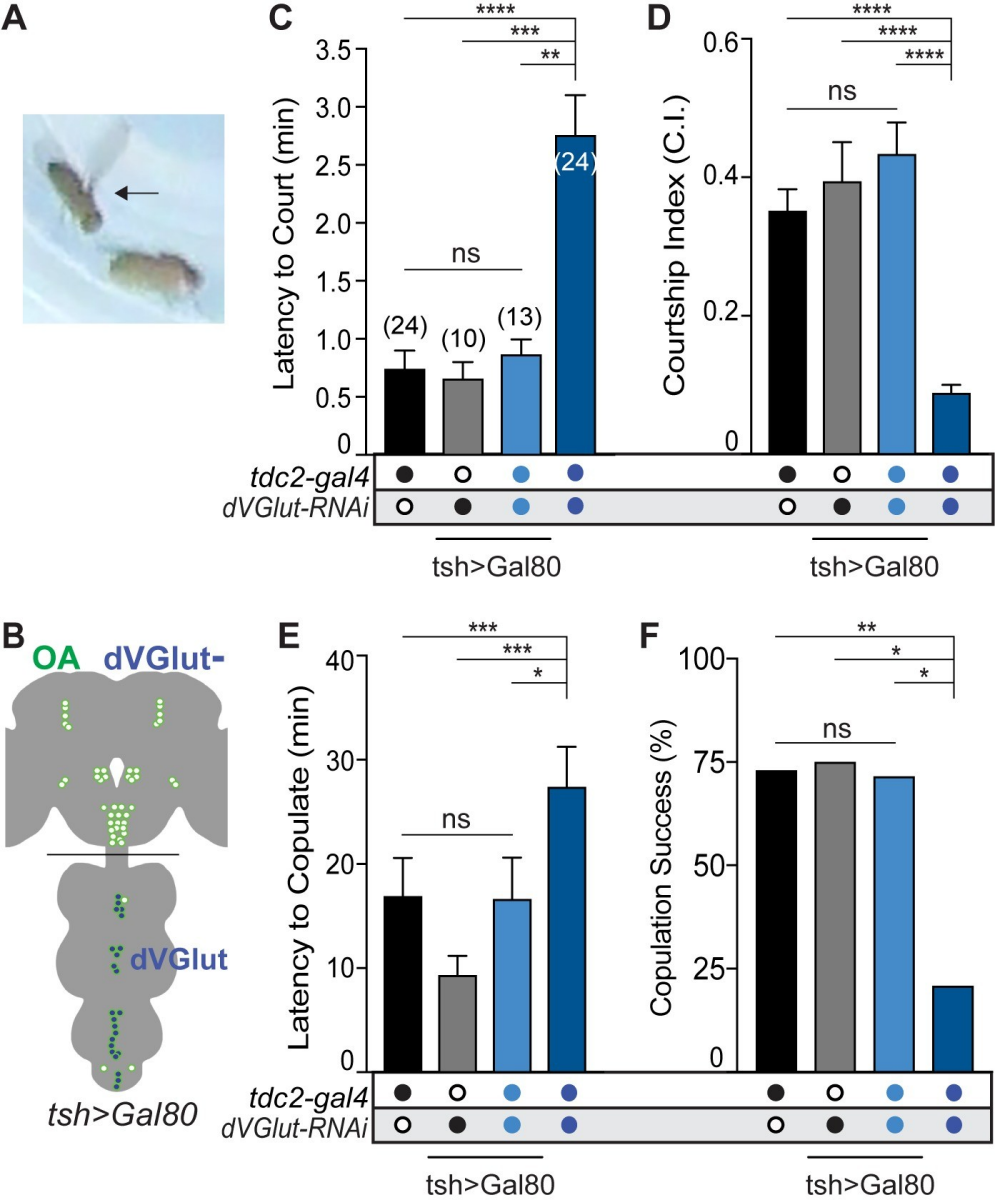
dVGLUT function is required in VNS OGNs for male-female courtship. **(A)** Male (arrow) to female courtship. **(B)** Schematic illustrating the addition of *tsh>Gal80* limits dVGLUT reduction to brain OGNs. **(C-F)** All parameters of male to female courtship were rescued by restoring glutamate function to OGNs within the VNC. **(C)** The latency to initiate courtship towards a female returned to control levels in males with reduced dVGLUT in brain OGNs. **(D)** The courtship index was restored to control levels in *tdc2-gal4/tsh>Gal80;dVGlut-RNAi* males. **(E)** *tdc2-gal4/tsh>Gal80;dVGlut-RNAi* males exhibited the same latency to copulation as controls. **(F)** The copulation success of males with a dVGLUT reduction in brain OGNs was not significantly different from controls. N values for each genotype located on panel A. All statistical tests are Kruskal-Wallis with Dunn’s multiple comparisons test, (*p<0.05, **p<0.01, ***p<0.001, ****p<0.0001.

With dVGlut function maintained in motor neurons, it was possible all aggressive behaviors would return to control levels. However, latency to initiate aggression remained longer in males with reduced dVGLUT in brain OGNs (*tdc2>tsh>Gal80>dVGlut-RNAi*) and lunge number remained lower when compared to controls (Fig 2I and 2J). Wing threat numbers were at levels lower than one control (Fig 2K) which likely reflects the incompleteness of dVGlut RNAi interference. In contrast, providing dVGLUT function in OGN VNS neurons restored intermale courtship to control levels (Fig 2L). Although total behavioral events by experimental males (lunges, wing threats, intermale courtship) per minute decreased, overall activity did not (S7 Fig) nor did male-female courtship (Fig 3). These results indicating GLU transport in brain OGNs is required to initiate aggression and for the lunge pattern itself may reflect deficits in the detection of male pheromones as we previously described for OA [43]. Specifically, the suppression of intermale courtship requires the function of three OA-FruM+ neurons located in the brain [55] and, aggression requires pheromonal information from Gr32a-expressing chemosensory neurons located in the mouth to OA SEZ neurons [43].

Finally, males with reduced dVGLUT in brain OGNs (*tdc2>tsh>Gal80>dVGlut-RNAi*) performed all measured male-female courtship parameters including latency to court, courtship index, latency to copulation and copulation success at levels indistinguishable from controls (Fig 3). Together, these results indicate dVGlut in OGNs is required in males both for aggression and courtship toward a female and at the behavioral level, the functional requirement for dVGLUT in OGN motor neurons vs. central brain neurons is spatially separable.

### Removal of glutamate in OGNs using the *B3RT-vGlut* conditional allele

**Fig 4:**
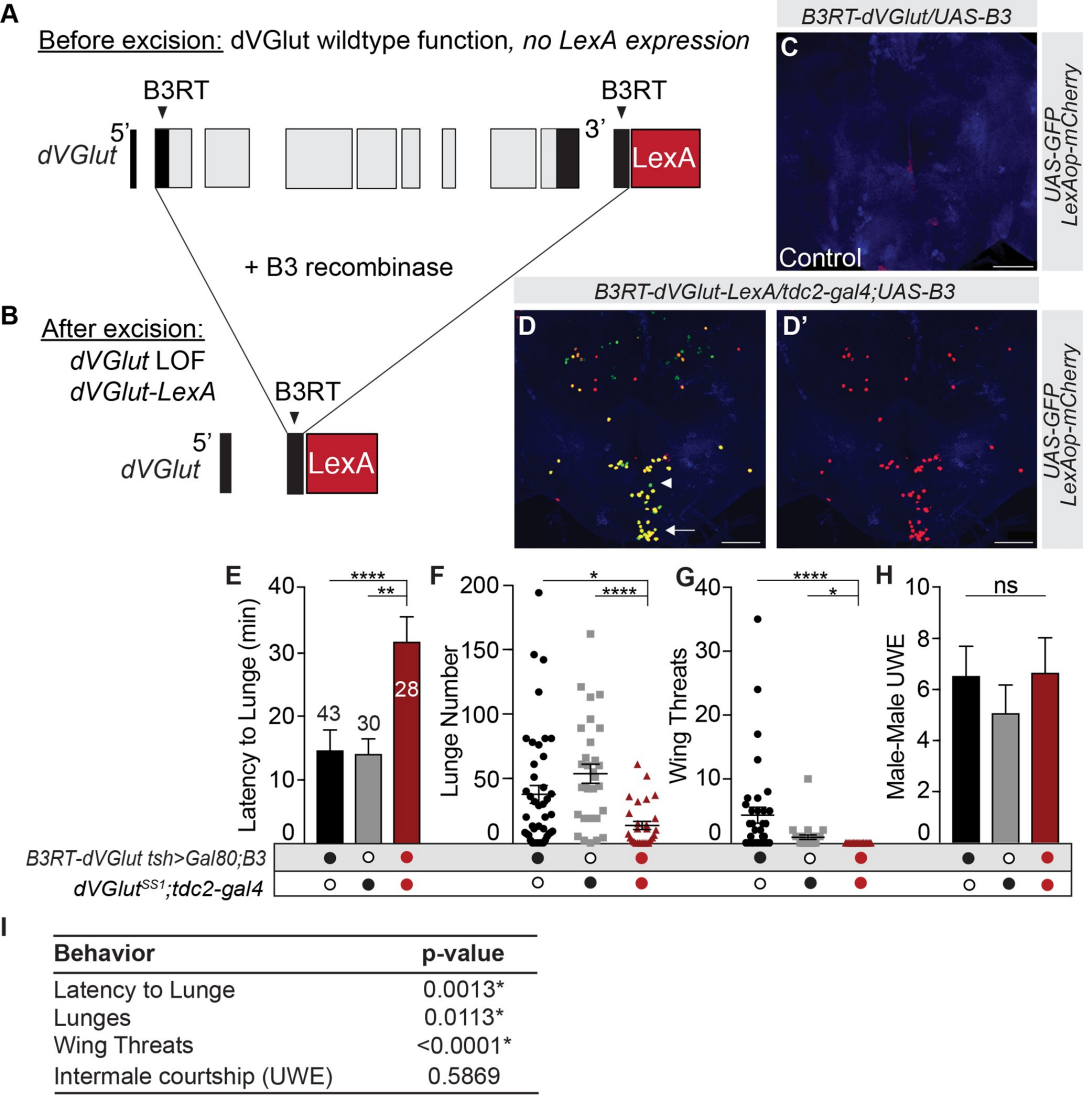
B3-mediated elimination of dVGLUT in OGNs reduces male aggression. **(A,B)** Schematic of the *B3RT-dVGlut-LexA* conditional allele. B3RTs flank dVGlut coding exons **(A)** and excise the entire dVGlut coding sequence in a specific subset of neurons upon expression of the B3 recombinase **(B)**. After excision, a *dVGlut* null loss-of-function allele and *dVGlut-LexA* driver is created **(B)**. **(C)** Control brain demonstrating without a source of Gal4-driven B3 recombinase, excision and therefore LexA expression does not occur. **(D-D’)** *tdc2-gal4* driven B3 recombinase-mediated excision effectively removes *dVGlut* resulting in *B3RT-LexA-*driven mCherry expression is in the majority of OA neurons (yellow). As expected, a few Tdc2+ neurons do not express dVGLUT (arrowhead, green). LexAop reporter expression that does not also show UAS expression may be observed as a result of excisions that occurred during development in former Tdc2+ neurons. **(E)** Latency to lunge increased in males lacking dVGLUT function (*B3RT-dVGlut tsh>Gal80*/*dVGlut*^*SS1*^*;UAS-B3*) in OGNs. **(F)** Males without dVGLUT function lunged significantly less when compared to controls. **(G)** Wing threat number decreased in experimental males. **(H)** No significant differences in male-male courtship. **(I)** Aggression is significantly reduced by the complete loss of dVGLUT in OGNs as compared to the RNAi-based dVGLUT reduction. All statistical tests are Kruskal-Wallis with Dunn’s multiple comparisons test, (*p<0.05, **p<0.01, ***p<0.001, ****p<0.0001. Error bars denote s.e.m. N values for each genotype, panel E.

The experiments above used two different approaches to reduce neurotransmitter levels, but not eliminate dVGLUT. To completely remove glutamate transporter function in OGNs, a conditional allele of *dVGlut*, *B3RT-dVGlut-LexA* (hereafter *B3RT-dVGlut*), was developed via genome editing. Genome edits to the *dVGlut* locus included flanking the dVGlut coding exons with B3 recombination target sites (B3RTs) [60] in the same orientation and inserting the coding sequences of the LexA transcription factor immediately downstream of the 3’ B3RT (Fig 4A). With *B3RT-dVGlut*, glutamate function can be temporally and spatially controlled using Gal4 drivers of interest to express the B3 recombinase that in turn catalyzes the *in vivo* excision of DNA between the B3RTs (Fig 4B). Two outcomes result after B3 recombinase-mediated excision; 1) a *dVGlut* null allele is generated solely in the neurons of interest, and 2) a *dVGlut-LexA* driver is created that allows visualization of glutamatergic neurons when a LexAop reporter is present.

To assess the functionality of *dVGlut* within the *B3RT-dVGlut* chromosome pre- and post-excision, the *B3RT-dVGlut* chromosome was crossed with the null allele, *dVGlut*^*SS1*^ (S1 Fig). In the absence of a Gal4 driver, *vGlut*^*SS1*^*/B3RT-vGlut* progeny are fully viable and no LexAop-driven reporter gene expression is detected (Fig 4C). In contrast, when B3 recombinase (*UAS-B3)* is expressed in the nervous system by the pan-neuronal driver, *n-syb-Gal4*, dVGLUT expression is eliminated and *vGlut*^*SS1*^*/B3RT-dVGlut;UAS-B3/n-syb-Gal4* progeny are inviable (data not shown). These results establish that the *B3RT-dVGlut* genome edits preserve dVGLUT function prior to excision, but after excision, as expected with removal of the entire dVGLUT protein-coding sequence, a *dVGlut* null allele is generated.

To verify the functionality of the *B3RT-dVGlut* chromosome in Tdc2+ neurons, we crossed *tdc2-gal4* with *B3RT-dVGlut;UAS-B3*. Following B3-mediated excision in Tdc2+ neurons, the resulting *dVGlut-lexA* driver is active in OGNs demonstrating the *dVGlut* coding region was removed. The excision of dVGlut and substitution with LexA in the adult nervous system was confirmed by co-localization of nuclear markers (Fig 4D and 4D’). This result provides additional confirmation the majority of Tdc2+ neurons are glutamatergic. In addition, nuclear reporters were used to confirm the loss of dVGLUT does not obviously alter OGN differentiation (S9 Fig).

To completely remove dVGLUT function, we used the *dVGlut*^*SS1*^ null allele in combination with the *B3RT-dVGlut* conditional null allele. Due to the requirements for GLU in OA-GLU motor neurons, we crossed the *tsh>Gal80* transgenes onto the *B3RT-dVGlut* chromosome. Males with homozygous null *dVGlut* mutations in brain OGNs were generated by driving B3 recombinase with *tdc2-gal4* (*dVGlut*^*SS1*^*/B3RT-dVGlut tsh>Gal80;UAS-B3/tdc2-gal4*). As expected, the complete loss of GLU in brain OGNs reduced male aggression. Specifically, the latency to initiate aggression increased, and lunge numbers decreased (Fig 4E and 4F). Not unexpectedly, the complete elimination of dVGLUT function resulted in aggression deficits significantly worse when compared to the RNAi approach (Fig 4I) including now a reduction in wing threat number (Fig 4G) which demonstrates an advantage in using the conditional null *B3RT-dVGlut* allele. Finally, and significantly, the number of inter-male wing extensions did not differ from controls (Fig 4H) nor from males with a reduction of *dVGlut* in brain OGNs (Fig 2K). In summation, the *dVGlut*^*SS1*^*/B3RT-dVGlut* null combination elegantly and independently validates the aggression phenotypes based on *dVGlut* RNAi-based reduction, demonstrates the applicability of a powerful new conditional genetic tool, and confirms that dVGLUT function in OGNs is not required to regulate intermale courtship.

### Reducing GLU by EAAT1 overexpression recapitulates the decrease in aggression

**Fig 5:**
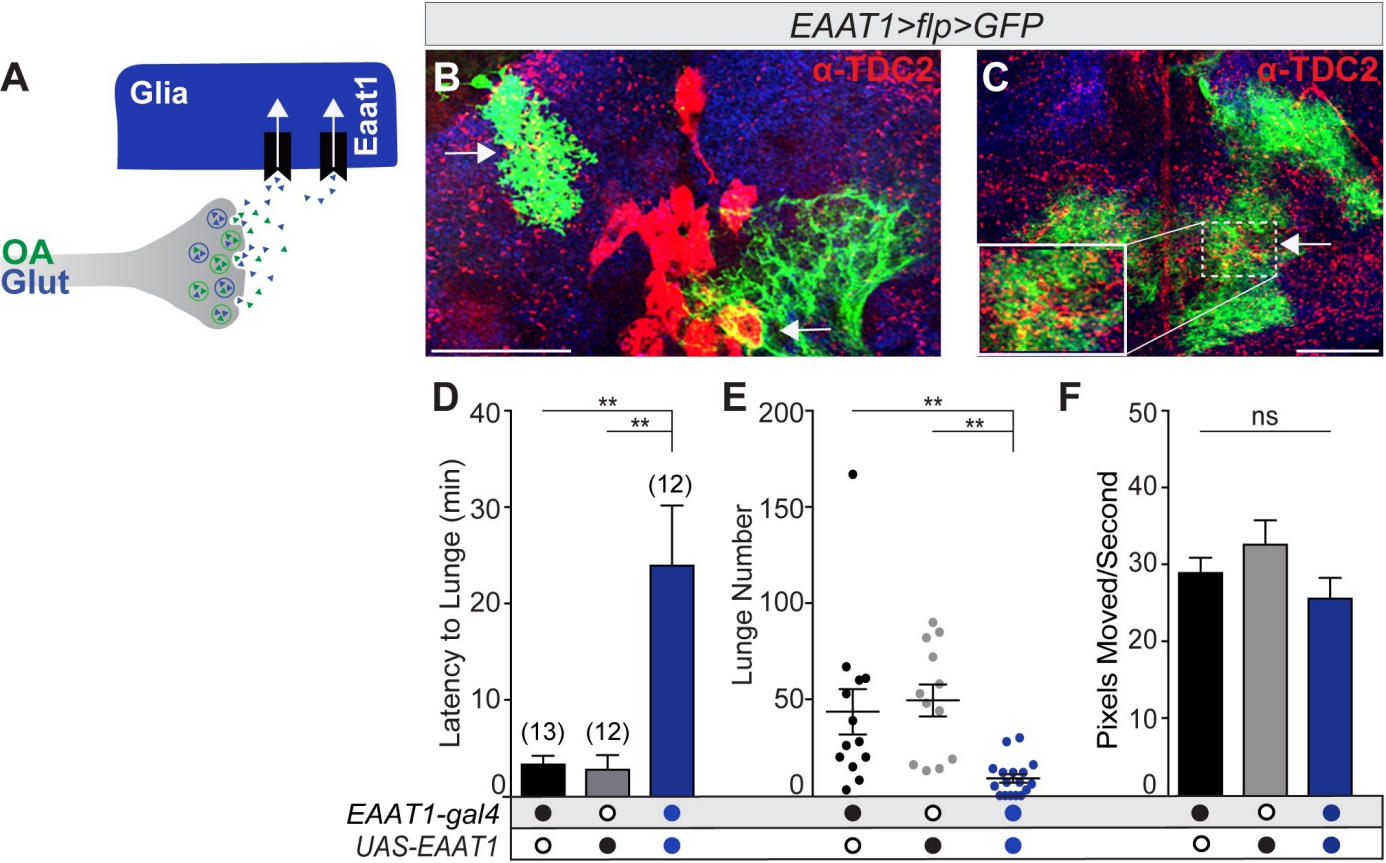
Reducing glutamate function through EAAT1 overexpression decreases male aggression. **(A)** Glutamate function was reduced by increasing EAAT1 expression in EAAT1-expressing glia. **(B, C)** GFP-expressing EAAT1 glia (*hs-flp; EAAT1-gal4/UAS>stop>CD8*:*GFP*) enwrap Tdc2+ neuron cell bodies (arrowhead) and endings (arrow). Higher magnification of dashed box in C. Scale bar = 30 um. **(D)** The latency to lunge by *EAAT1>Eaat1* males was increased as compared to controls. **(E)** A decrease in lunge number was exhibited by *EAAT1>Eaat1* males as compared to controls. **(F)** Locomotor activity during the aggression assay did not differ. All statistical tests are Kruskal-Wallis with Dunn’s multiple comparisons tests. N values for each genotype are in panel D.

At this point, GLU function within OGNs has been altered by reducing glutamate transport into synaptic vesicles. Whether the aggression phenotypes of OGN dVGLUT mutant males are due to deficits in the concentration of GLU into synaptic vesicles, the packaging of OA, or a reduction of released GLU is not clear. After release, glutamate is rapidly removed from synapses by excitatory amino acid transporters (EAATs) [61, 62]. Therefore, to reduce GLU signaling after release, we increased expression of the only high-affinity glutamate transporter in *Drosophila*, EAAT1 (Fig 5A) [63, 64].

EAAT1 is expressed in glia throughout the nervous system [64]. By examining 2–10 individual EAAT1-GFP clones in ~40 brains, we determined OGN neuronal cell bodies and arborizations are consistently enmeshed by EAAT1-expressing glia (Fig 5B and 5C). To reduce glutamate signaling after release, EAAT1 expression was increased via a transgene (*EAAT1-gal4;UAS-EAAT1*). While a loss of EAAT1 impairs larval movement [65], overexpression of EAAT1 has been used in adult long-term memory formation assays which requires locomotion [66]. Similar to the dVGLUT loss-of-function results above, the aggressive behavior of males with reduced GLU signaling by EAAT1 overexpression (*EAAT1-gal4;UAS-EAAT1*) was altered in two parameters: the latency to initiate lunging increased and lunge number decreased (Fig 5D and 5E). Locomotor activity during the aggression assay did not differ (Fig 5F). Although future experiments will be needed to determine if the promotion of aggression requires dVGLUT packaging of OA in synaptic vesicles and OGN glutamate signaling to downstream targets, results from this section support the hypothesis that OGN-mediated aggression requires GLU.

### OA and Glu signal to a shared aggression-promoting circuit

**Fig 6:**
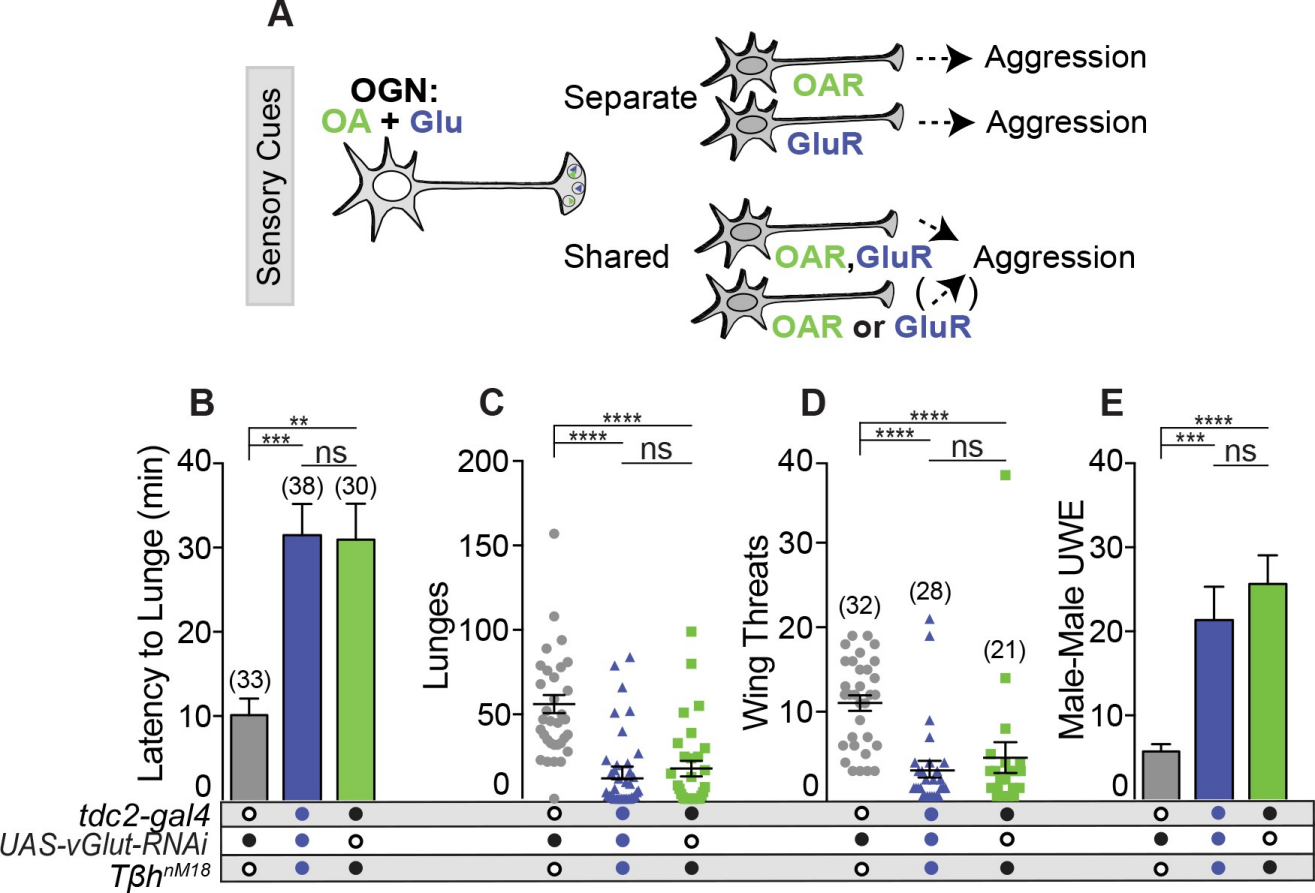
OA and Glu signal to a shared aggression-promoting circuit. **(A)** OGNs could signal to separate aggression-promoting circuits (resulting in aggression deficits greater than the single mutant) or to a shared or converged circuit. **(B-E)** dVGlut was reduced in OGNs of *Tβh*^*M18*^ males (*Tβh*^*M18*^*;tdc2>dVGlut-RNAi)*. **(B)** Latency to lunge increased in *Tβh*^*M18*^*;tdc2>dVGlut-RNAi* males compared to the transgenic control but not *Tβh*^*M18*^ males. **(C)** Lunge number by males with reduced dVGLUT and lacking OA was not significantly different than *Tβh*^*M18*^ males. **(D)** *Tβh*^*M18*^*;tdc2>dVGlut-RNAi* males displayed lower wing threat numbers compared to the transgenic control but not *Tβh*^*M18*^ males. **(E)** Males with reduced dVGLUT and lacking OA (blue column) displayed an increase in inter-male courtship at levels higher than the control but not significantly different from *Tβh*^*M18*^ mutants (green column). All statistical tests are Kruskal-Wallis with Dunn’s multiple comparisons test, (*p<0.05, **p<0.01, ***p<0.001, ****p<0.0001. Error bars denote s.e.m.

If Glu and OA convey signals to separable aggression-promoting circuits, a loss of both neurotransmitters would reduce aggression greater than the loss of either alone (Fig 6A). If, however, Glu and OA signal to a shared circuit or circuits that converge, a loss of both transmitters would reduce aggression to the same levels as the loss of one alone. To address this question, we incorporated the previously described null allele *Tβh*^*nM18*^ [67] and generated *Tβh*^*nM18*^*;tdc2>dVGlut-RNAi* males. Additive deficits did not occur when males without OA and dVGLUT in OGNs were compared to males lacking only OA (Fig 6B–6D) indicating that both signals, at least partially, converge onto a shared aggression-promoting pathway.

*Tβh*^*nM18*^*;tdc2>vGlut-RNAi* males displayed levels of male-male courtship that are not significantly different from *Tβh*^*nM18*^ males (blue column, Fig 6E). This result further supports previously published data that OA is required to suppress intermale courtship [43, 55, 56]. Here, increased levels of inter-male courtship due to the absence of OA supersedes or relieves the lack of UWE due to a reduction in dVGlut function (Fig 2). At this point, it is possible the UWE phenotype occurs via OA-modulated circuitry that involves other neurotransmitters [56] or the actions of OA occur at spatially distinct locations.

### Spatial segregation of VMAT and dVGLUT within OGN

**Fig 7:**
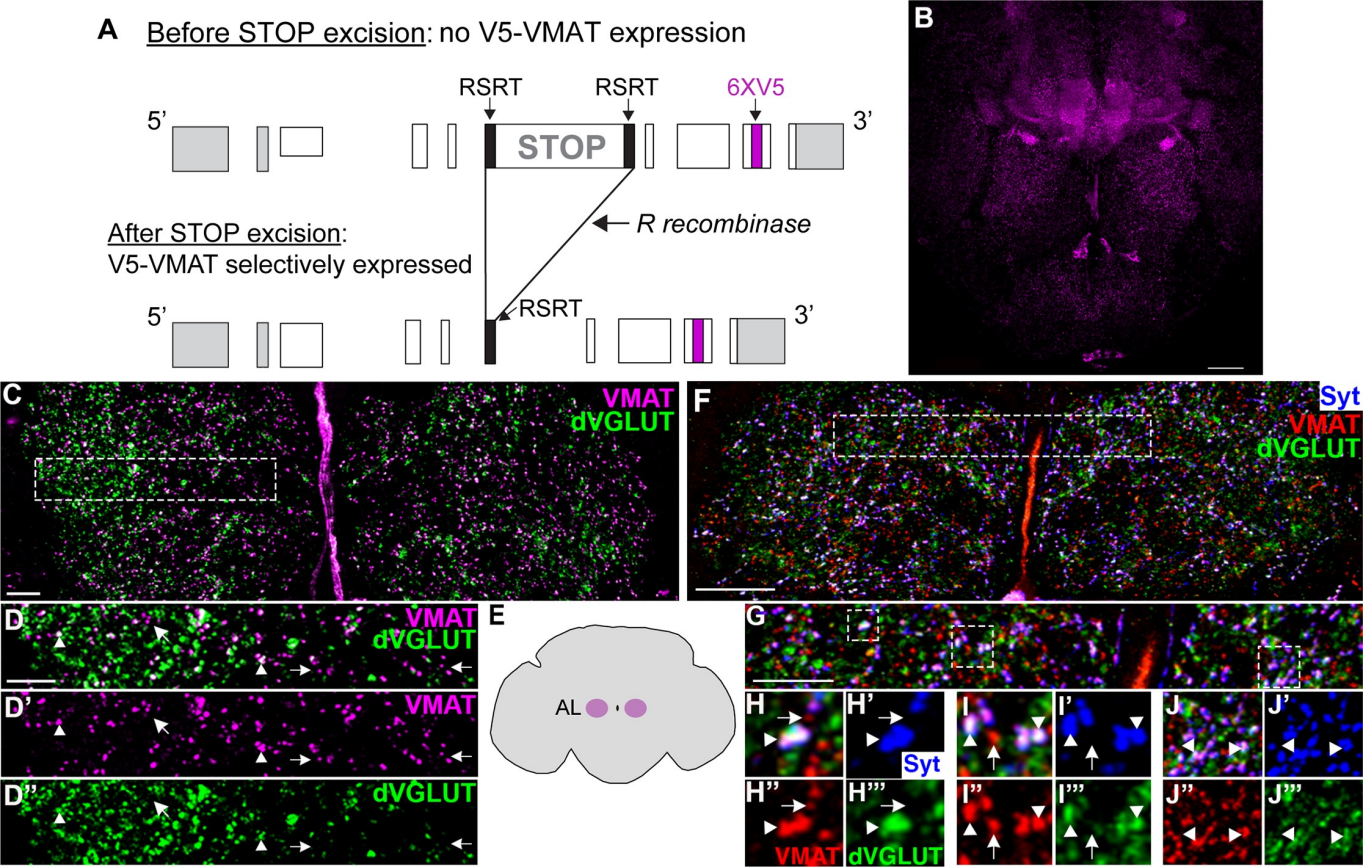
Spatial segregation of VMAT and dVGLUT within OGNs. **(A)** Schematic of the *RSRT>STOP>RSRT-6XV5-VMAT* conditional allele. RSRTs flank a STOP cassette inserted between VMAT coding exon 5 and 6. Upon Gal4-driven expression of the R recombinase enzyme, the STOP cassette is excised and V5-tagged VMAT expression under control of the endogenous promoter is expressed. **(B)** Representative brain showing V5-VMAT expression in OGNs after excision by *tdc2-dVGlut-gal4* driven R recombinase. The brain is labeled with anti-V5 (magenta) and mAb dVGLUT (green in panels C,D). Scale bar is 30 μm. **(C)** Higher magnification of the antennal lobe region showing dVGLUT expression (green) with V5-VMAT (magenta). Scale bar is 10 μm. **(D)** The region in the dashed box in C showing puncta with dVGLUT and V5-VMAT colocalization (arrowheads) and puncta with only V5-VMAT (arrows). **(E)** Schematic showing the regions of the brain that are depicted in C and F. **(F)** Antennal lobe region of a representative brain with a synaptic marker incorporated (*UAS-synaptotagmin;HA*, *tdc2-dVGlut split gal4/UAS-R RSRT-STOP-RSRT-6XV5-vMAT)*. The brain is labeled with anti-HA (blue), anti-V5 (magenta), and mAb dVGLUT (green). Scale bar is 20 μm. **(G-J”’)** Higher magnification of the SEZ region of the AL in F showing dVGLUT expression (green), V5-VMAT (red), and Syt:HA (blue). Arrowheads indicate puncta with dVGLUT, V5-VMAT and Syt:HA and arrows indicate puncta with only V5-VMAT and Syt:HA. The stack for panels C and D contains two optical sections at 0.45 μm. Stacks for panels G-J contain 7 optical sections at 0.5 μm.

To compare localization of the two transporters within OGNs, we generated a conditionally expressible epitope-tagged version of VMAT, *RSRT>STOP>RSRT-6XV5-VMAT*, via genome editing. *RSRT>STOP>RSRT-6XV5-VMAT* has two insertions: 1) a STOP cassette between VMAT coding exons 5 and 6 and, 2) six in-frame tandem copies of a V5 epitope tag within exon 8 which is common to both VMAT-A and VMAT-B isoforms (Fig 7A). The effectiveness of the STOP cassette is confirmed by the lack of V5 expression prior to STOP cassette excision by Gal4-driven R recombinase (S11 Fig) and the effectiveness of the epitope multimerization strategy has also been determined [68]. The conditionality of the *RSRT>STOP>RSRT-6XV5-VMAT* allele permits visualization of VMAT in subsets of neurons at expression levels driven by the endogenous promoter.

**Fig 8:**
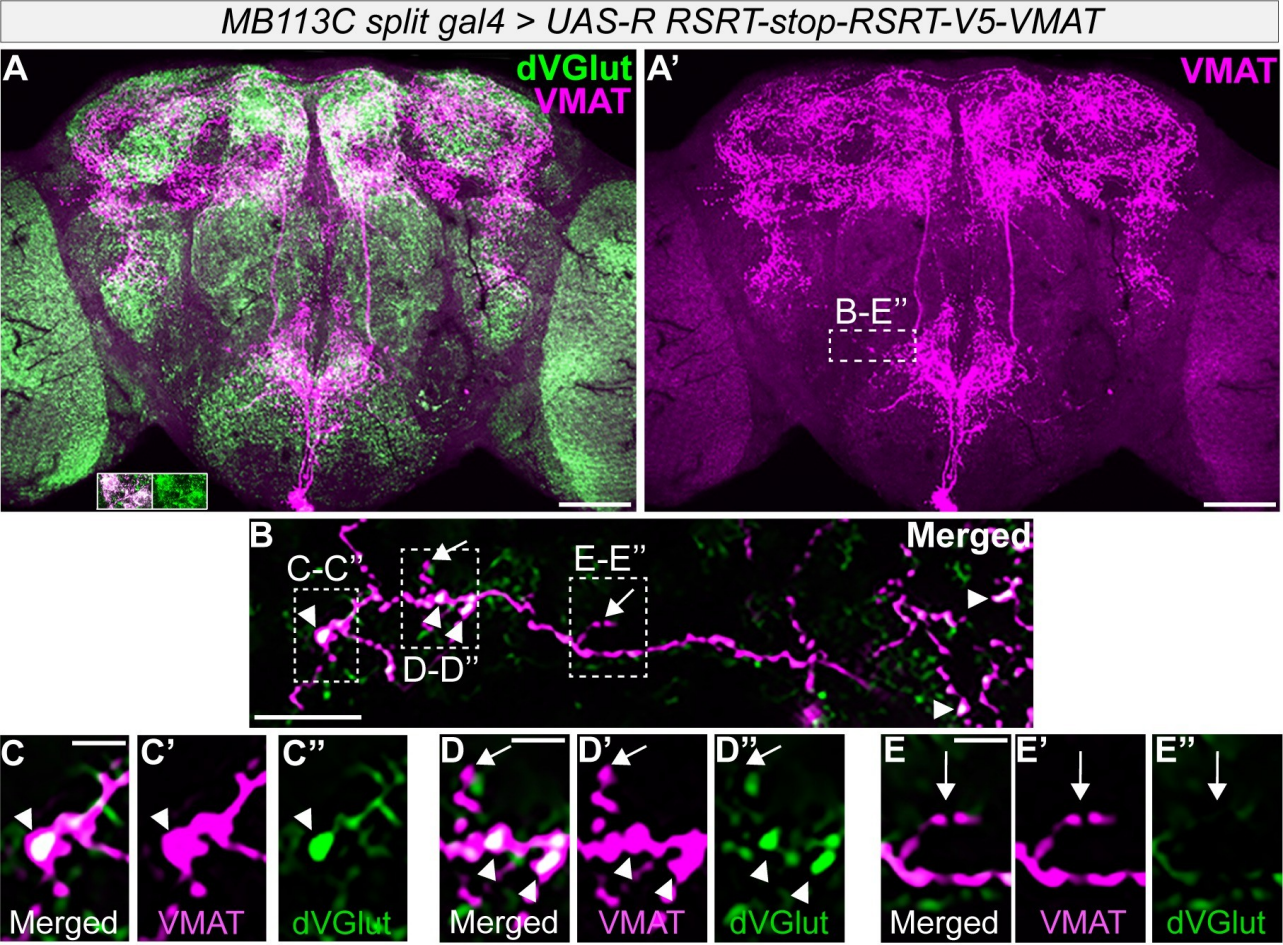
Spatial segregation of VMAT and dVGLUT within two OGNs. **(A-A’)** Representative brain showing V5-VMAT expression in two OGNs after excision by *MB113C-split-gal4* driven R recombinase. The brain is labeled with anti-V5 (magenta) and mAb dVGLUT (green). Scale bar is 50 μm. The inlet in A which is from a separate brain demonstrates this OA neuron driver also expresses dVGLUT (green). **(B-E)** Higher magnification of the SEZ boxed region in A’. Arrowheads point to puncta with V5-VMAT and dVGLUT, arrows indicate V5-VMAT only puncta. Scale bar is 10 μm. **(C-E)** The regions in the dashed boxes in B showing puncta with dVGLUT and V5-VMAT colocalization (arrowheads) and puncta with only V5-VMAT (arrows). Panels B-E contain stacks of four optical sections at 0.45 μm. Scale bar for panels C-E is 5 μm.

To focus on transporter distribution within OGNs, we expressed *RSRT>STOP>RSRT-6XV5-VMAT* under control of the split Gal4 combination of *tdc2-Gal4-AD* and *dVGlut-Gal4-DBD (tdc2-dVGlut-gal4)* which drives expression in OGNs (Fig 7B, S6C–S6F Fig). V5-VMAT was visualized in *tdc2-dVGlut-gal4; V5-VMAT UAS-R* by an antibody to V5 and dVGLUT using mAb dVGLUT (S10 Fig). Fig 7C illustrates that as expected, a large fraction of the V5-VMAT puncta in the AL or SEZ (S11 Fig) either co-localize with dVGLUT or are in close proximity (arrowheads). High resolution images in Fig 7D and 7H, however, reveal V5-VMAT puncta without dVGLUT (arrows). As OA can be found in SVs as well as LDCVs [69, 70], we incorporated a synaptic marker (*UAS-Synaptotagmin (Syt)*:*HA*) and re-examined V5-VMAT and dVGLUT expression in the AL and SEZ (Fig 7F, S11D Fig). We found V5-VMAT puncta that either co-localize or are in close proximity to Syt:HA and dVGLUT (Fig 7F–7J, S11D–S11H Fig). While the behavioral significance of potential OA synaptic release on aggression circuitry remains to be determined, previous work has demonstrated amine-dependent behaviors can be altered by shifting the balance of OA release from SVs to LDCVs [70]. In addition, as mentioned above, we have previously shown that three OA-FruM^+^ neurons are required to suppress intermale courtship and recent work has identified a small subset of OA receptor OAMB-expressing neurons that when silenced, decrease aggression and increase intermale courtship [56]. The SEZ areas of V5-VMAT and dVGLUT puncta highlighted in Figs 7 and 8 are consistent with projections made by OA-FruM^+^ neurons which are also OGNs (S12 Fig) raising the possibility of distinct OA and GLU inputs to key downstream targets.

Due to the large number of *tdc2-dVGlut-gal4* neurons, we repeated the experiment using the OA-specific *MB113C-split-gal4* to drive V5-VMAT in ~2 OGNs (Fig 8A and 8B) [71]. Fig 8C illustrates that as expected, many V5-VMAT puncta in the SEZ either co-localize with dVGLUT or are in close proximity (arrowheads). High resolution images in Fig 8D and 8H, however, indicate small, but distinct regions that contain V5-VMAT puncta without dVGLUT (arrows). Within the areas of dVGLUT and V5-VMAT possible colocalization, this level of analysis does not indicate whether the two transporters segregate into adjacent but distinct puncta, nor are questions of transporter colocalization on the same vesicles addressed. Nevertheless, our results demonstrate that within OGNs, V5-VMAT and dVGLUT puncta can differ in localization suggesting the aggression vs. intermale courtship phenotype differences may be due to spatial differences in signaling by glutamate and octopamine.

## Discussion

Addressing the functional complexities of ‘‘one neuron, multiple transmitters” is critical to understanding how neuron communication, circuit computation, and behavior can be regulated by a single neuron. Over many decades, significant progress has been made elucidating the functional properties of neurons co-expressing neuropeptides and small molecule neurotransmitters, where the neuropeptide acts as a co-transmitter and modulates the action of the neurotransmitter [5, 6, 72]. Only recently have studies begun to examine the functional significance of co-transmission by a fast-acting neurotransmitter and a slow-acting monoamine.

In this study, we demonstrated that OA neurons express dVGLUT and utilized a new genetic tool to remove dVGLUT in OA-glutamate neurons. Quantifying changes in the complex social behaviors of aggression and courtship revealed that dVGLUT in brain OGNs is required to promote aggressive behavior and a specific behavioral pattern, the lunge. In contrast, males deficient for dVGLUT function do not exhibit an increase in inter-male courtship. These results establish a previously undetermined role for dVGLUT in OA neurons located in the adult brain and reveal glutamate uncouples aggression from inter-male courtship. It has been suggested that classical neurotransmitters and monoamines present in the same neuron modulate each other’s packaging into synaptic vesicles or after release via autoreceptors [9, 49, 73–75]. For example, a reduction of dVGLUT in DA-glutamate neurons resulted in decreased AMPH-stimulated hyperlocomotion in *Drosophila* and mice suggesting a key function of dVGLUT is the mediation of vesicular DA content [12, 49, 76]. In this study, the independent behavioral changes suggests enhancing the packaging of OA into vesicles is not the sole function of dVGLUT co-expression and suggests differences in signaling by OA from OGNs on courtship-related circuitry.

Co-transmission can generate distinct circuit-level effects via multiple mechanisms. One mechanism includes spatial segregation; the release of two neurotransmitters or a neurotransmitter and monoamine from a single neuron occurring at different axon terminals or presynaptic zones. Recent studies examining this possible mechanism have described; (i) the release of GLU and DA from different synaptic vesicles in midbrain dopamine neurons[15, 77] and (ii) the presence of VMAT and VGLUT microdomains in a subset of rodent mesoaccumbens DA neurons[78]. In this study, we expressed a new conditionally expressed epitope-tagged version of VMAT in OGNs and visualized endogenous dVGLUT via antibody labeling. Within OGNs, the colocalization of VMAT and dVGLUT puncta was not complete suggesting the observed behavioral phenotype differences may be due to spatial differences in OA signaling.

A second mechanism by which co-transmission may generate unique functional properties relies on activating distinct postsynaptic receptors. In *Drosophila*, recent work has identified a small population of male-specific neurons that express the alpha-like adrenergic receptor, OAMB, as aggression-promoting circuit-level neuronal targets of OA modulation independent of any effect on arousal[56] and separately knockdown of the *Rdl* GABAa receptor in a specific *doublesex+* population stimulated male aggression [79]. Future experiments identifying downstream targets that express both glutamate and octopamine receptors would be informative, as well as using additional split-Gal4 lines to determine if segregation of transporters is a hallmark of the majority of OGNs. Finally, a third possible mechanism is Glu may be co-released from OGNs and act on autoreceptors to regulate presynaptic OA release (reviewed in [75]).

Deciphering the signaling complexity that allows neural networks to integrate external stimuli with internal states to generate context-appropriate social behavior is a challenging endeavor. Neuromodulators including monoamines are released to signal changes in an animal’s environment and positively or negatively reinforce network output. In invertebrates, a role for OA in responding to external chemosensory cues as well as promoting aggression has been well-established [43, 47, 56, 80–83]. In terms of identifying specific aggression circuit-components that utilize OA, previous results determined OA neurons directly receive male-specific pheromone information [43] and the aSP2 neurons serve as a hub through which OA can bias output from a multi-functional social behavior network towards aggression[56]. The ability of OA to bias behavioral decisions based on positive and negative reinforcement was also recently described for food odors [84]. In vertebrates, it has been proposed that DA-GLU cotransmission in the NAc medial shell might facilitate behavioral switching [85]. Our finding that the majority of OA neurons are glutamatergic, suggests that the complex social behavior of aggression may rely on small subsets of neurons that both signal the rapid temporal coding of critical external stimuli as well as the frequency coding of such stimuli resulting in the enhancement of this behavioral network. One implication of our finding regarding the separable OA-dependent inhibition of inter-male courtship is the possibility of identifying specific synapses or axon terminals that when activated gate two different behavioral outcomes. A second implication is that aggressive behavior in other systems may be modified by targeting GLU function in monoamine neurons.

Finally, monoamine-expressing neurons play key roles in human behavior including aggression and illnesses that have an aggressive component such as depression, addiction, anxiety, and Alzheimer’s [86, 87]. While progress is being made in addressing the functional complexities of dual transmission, the possible pathological implications of glutamate co-release by monoamine neurons remains virtually unknown. Analyzing the synaptic vesicle and release properties of monoamine-glutamate neurons could offer new possibilities for therapeutic interventions aimed at controlling out-of-context aggression.

## Methods

### Drosophila husbandry and stocks

All flies were reared on standard cornmeal-based fly food. Unless noted otherwise, during developmental and post-eclosion, flies were raised at 25°C, ~50% humidity and a 12:12hr light-dark cycle (1400±200 lx white fluorescent light) in humidity and temperature-controlled incubators. A list of stocks can be found in S1 Data.

### Aggression assays

Male pupae were isolated and aged individually in 16 x 100mm borosilicate glass tubes containing 1.5ml of standard food medium as previously described [88]. A dab of white or blue acrylic paint was applied to the thorax of two-day old males under CO_2_ anesthesia for identification purposes. Flies were returned to their respective isolation tubes for a period of at least 24 hours to allow recovery. For aggression testing, pairs of 3–5 day old, socially naïve adult males were placed in 12-well polystyrene plates (VWR #82050–930) as described previously [43]. All assays were run at 25°C and ~45–50% humidity levels.

### Scoring and statistics

All aggression was assayed within first two hours of lights ON time (Zeitgeber hours 0–2) and scored manually using iMovie version 8.0.6. Total number of lunges, wing threats, and unilateral wing extensions were scored for a period of 30 minutes after the first lunge according to the criteria established previously [43, 88]. The time between the aspiration of the flies into the chamber and the first lunge was used for calculating the latency to lunge. Male-male courtship was the number of unilateral wing extensions (singing) followed by abdomen bends or repeated wing extensions. All graphs were generated with Graphpad Prism and Adobe Illustrator CS6. For data that did not meet parametric assumptions, Kruskal-Wallis Test with Dunn’s multiple comparison was used unless otherwise specified. A standard unpaired t-test was performed in the case of only two comparisons and a modified chi-square test to compare copulation success.

### Activity levels

Activity levels were measured by tracking the flies in each assay using the OpenCV module in the Python programming language to analyze the video and then output XY-coordinate and distance data. The distance traveled was calculated for each fly by determining the starting location followed by the second location after a 250-ms time interval and then taking the sum of the distance traveled in each interval. To calculate pixels moved per second, the distance data was divided by the total time spent tracking.

### Immunohistochemistry

Adult male dissected brains were fixed in 4% paraformaldehyde (Electron Microscopy Sciences) for 25 minutes and labeled using a modification of protocols previously described [55]. The following primary antibodies were used: anti-bruchpilot (mAb nc82, 1:30, Developmental Studies Hybridoma Bank developed under the auspices of the NICHD and maintained by the Department of Biology, University of Iowa (Iowa City, IA).), monoclonal rabbit anti-GFP (1:200, Molecular Probes), rat anti-HA 3F10 (1:100, Roche), mAb dVGLUT (1:15), anti-TβH (1:400, [89]), rat anti-V5 (1:200, Biorbyt), and rabbit anti-TDC2 (1:100, Covalab). Secondary antibodies conjugated to Alexa 488, Alexa 594, or Alexa 647 (Molecular Probes) were used at a concentration of 1:200. Labeled brains were mounted in Vectashield (Vector Labs, #H1000). Images were collected on an Olympus Fluoview FV1000 laser scanning confocal mounted on an inverted IX81 microscope and processed using ImageJ (NIH) and Adobe Photoshop (Adobe, CA).

### qPCR

Total RNA from ~40 heads using Direct-zol RNA Miniprep Pluskit (Zymo Research)and treated with DNase I per the manufacturer’s protocol. RNA concentrations were measured with a ND-1000 nanodrop spectrometer. Reverse transcription was accomplished using iScript cDNA Synthesis kit (Bio-Rad Laboratories). RT-PCR was performed using 300 ng cDNA added to iTaq Universal SYBR Green Supermix (Bio-Rad Laboratories) and primers in a 20 μL reaction volume. All samples were run in triplicate using a Stratagene Mx3005P qPCR System(Agilent Technologies). Expression of *ribosomal protein 49* (*Rp49*) was used as the reference control to normalize expression between genotypes. Expression levels were determined using the ΔΔCT method and results from control (*UAS-dVGlut-RNAi/+*) and experimental (*nsyb-Gal4/UAS-dVGlut-RNAi*) groups were normalized relative to a transgenic control (*nsyb-Gal4/+*). The following primers were used: Rp49 Forward: 50-CATCCGCCCAGCATACAG-3’ Rp49 Reverse: 5’-CCATTTGTGCGACAGCTTAG-3’ dVGlut Forward: 5’-GCACGGTCATGTGGTGATTTG-3’ dVGlut Reverse: 5’-CCAGAAACGCCAGATACCATGG-3’. Primer designs for all Rp49 and dVGlut primers used have been described previously [12].

### Construction of *20XUAS-His2A-GFP*, *13XLexAop2-His2B-mCherry* and *20XUAS-R*

The *20XUAS-His2A-GFP*, *13XLexAop2-His2B-mCherry*, and *20XUAS-R* expression clones were assembled using Gateway MultiSite LR reactions as previously described[90] and as indicated in S2 Table. The *L1-20XUAS-DSCP-L4* and *L1-13XLexAop2-DSCP-L4* entry clones contain 20 copies of UAS and 13 copies of LexAop2 upstream of the *Drosophila* synthetic core promoter (DSCP) [91], respectively. The *R4-His2A-R3* and *R4-His2B-R3* entry clones were generated as previously described [90] using genomic DNA as templates. The *L3-GFP-L2* entry clone was generated from template *pJFRC165*[60] except the PEST sequence is omitted. The *L3-GFP-L2* and *L3-mCherry-HA-L2* entry clones were previously described [92]. The *L1-20XUAS-DSCP-R5* entry clone was previously described [90]. The *pDESTp10aw* destination vector was previously described[93]. Injections were performed by Bestgene, Inc.

### Construction of *UAS-B3*

B3 recombinase derived from pJFRC157 [60] was PCR amplified using primers designed to add the syn21 translational enhancer sequence [94] and remove the PEST domain. The verified PCR product was cloned into pENTR (Invtrogen) and subsequently transferred to pBID20xUAS, a derivative of the pBID vector [95] with 20 copies of the UAS binding sequence. Injection of *UAS-B3* was performed by Genetivision into landing site VK31.

### Generation of *B3RT-vGlut*

The *B3RT-dVGlut-LexA* chromosome was generated via CRISPR/Cas9 genome editing. Both guide RNAs were incorporated into pCFD4 using previously described methods [96] to produce the double guide RNA plasmid *pCFD4-vGlut1*. The donor plasmid *B3RT-dVGlut-LexA* used the *pHSG298* backbone (Takara Bio) and was generated using NEBuilder HiFi (New England Biolabs). The complete annotated sequence of *B3RT-dVGlut-LexA* is shown in Supplementary Information. *pCFD4-vGlut1/B3RT-dVGlut-LexA* injections were performed by Bestgene, Inc.

To assess the functionality of *dVGlut* on the *B3RT-dVGlut* chromosome pre- and post-excision, the *B3RT-dVGlut* chromosome was crossed with the homozygous lethal *dVGlut* null allele, *dVGlut*^*SS1*^ in the presence and absence of the pan-neuronal driver *n-syb-Gal4*. In the absence of a Gal4 driver, *dVGlut*^*SS1*^*/B3RT-dVGlut* progeny are fully viable and no LexAop-driven reporter gene expression is detected (Fig 2). When B3 recombinase (*UAS-B3)* is expressed in the nervous system by *n-syb-Gal4*, *dVGlut*^*SS1*^*/B3RT-dVGlut;UAS-B3/n-syb-Gal4* progeny are inviable, therefore after excision, as expected with removal of the entire dVGlut protein-coding sequence, a *dVGlut* null allele results.

### Generation of *dVGlut*^*SS1*^

The *dVGlut*^*SS1*^ allele was generated by CRISPR/Cas9 genome editing with the same guide RNAs used to generate *B3RT-dVGlut LexA*. *dVGlut*^*SS1*^ was identified based on failed complementation with the existing *dVGlut*^*2*^ allele[97]. Sequencing of PCR products from this allele indicated a deletion of 2442bp that includes dVGlut amino acids 53–523. Genomic DNA sequence at the breakpoints of the *dVGlut*^*SS1*^ allele are indicated with the deleted region in bold: GGACCAGGCG**GCGGCCACGC**. . . . . .**AACCTCCGGC**CGAGGAGCAA.

### Generation of the *RSRT-STOP-RSRT-6XV5-vMAT* chromosome

*RSRT-STOP-RSRT-6XV5-vMAT* was generated via CRISPR/Cas9 genome editing. Both upstream guide RNAs were incorporated into pCFD4-vMAT1 and both downstream guide RNAs were incorporated into pCFD4-vMAT2 as previously described [96]. The *RSRT-STOP-RSRT-6XV5-vMAT* donor plasmid used the *pHSG298* backbone (Takara Bio) and was generated using NEBuilder HiFi (New England Biolabs). The complete annotated sequence of *RSRT-STOP-RSRT-6XV5-vMAT* is shown in Supplementary Information. *pCFD4-vMAT1/pCFD4-vMAT2/RSRT-STOP-RSRT-6XV5-vMAT* injections into the *nos-Cas9* strain *TH_attP2*[98] were performed by Bestgene, Inc.

The R and B3 recombinases from yeast recognize sequence-specific recombination target sites, RSRTs and B3RTs, respectively [60]. These recombinases are highly efficient and highly specific as they exhibit virtually no cross-reactivity with each other’s recombinase target sites. When pairs of recombinase target sites are in the same orientation, as is the case for both *B3RT-vGlut-LexA* and *RSRT-STOP-RSRT-6XV5-vMAT*, the recombinases catalyze excision of the intervening DNA and leave behind a single recombinase target site.

### dVGlut antibody

Drosophila anti-dVGLUT mouse monoclonal antibodies (10D6G) were generated (Life Technologies Europe) using the C-terminal peptide sequence TQGQMPSYDPQGYQQQ of dVGLUT coupled to KLH.

## Supporting information

S1 Fig Verification of mAb dVGLUT specificity using the null *dVGlut*^*SS1*^ allele. **(A)** dVGLUT expression detected by mAb dVGLUT in a heterozygous *yw*, *dVGlut*^*SS1*^*/+* late stage embryo. **(B)** dVGLUT expression is not detectable by mAb dVGLUT in a homozygous *yw*, *dVGlut*^*SS1*^*/ dVGlut*^*SS1*^ late stage embryo.
(TIF)

S2 Fig Multiple optical sections from *dVGlut>dsRed* male brains labeled with anti-Tβh. **(A-B)** Although the Tβh shows weaker immunoreactivity than the anti-Tdc2 antibody, Tβh is mainly detected in *dVGlut>dsRed* neurons at dorsal and ventral positions (A’, A”, B’ and B”). Scale bar = 20 μm.
(TIF)

S3 Fig **(A-A’)** Schematic showing the regions (boxes) of the VNS imaged in panels B and C. **(B-C)** A male *dVGlut>dsRed* adult VNS labeled with anti-Tdc2. The majority of dVGLUT+ neurons within the thoracic VNS **(B)** and abdominal VNS **(C)** express Tdc2 with a few exceptions (arrows). Scale bar = 10 μm.
(TIF)

S4 Fig **(A)** Schematic showing the regions imaged in panels B and C (colored boxes). **(B-C)** The majority of OA neurons within the PENP **(B)** and SEZ **(C)** regions co-express dVGLUT as visualized in a male *tdc2>dsRed* adult brain labeled with anti-dVGLUT. Scale bar = 10 μm.
(TIF)

S5 Fig **(A)** *dVGlut* transcript levels were decreased in *n-syb-gal4>dVGLUT-RNAi* males as compared to the *n-syb-gal4* control (n = 3; p<0.01). **(B-C)** Representative images of ventral sections of the SEZ from a *tdc2-gal4>dVGLUT-RNAi;UAS-dsRed* male brain labeled with anti-Tdc2. OGN differentiation as measured by Tdc2 expression is not altered by a reduction of dVGLUT. Scale bar = 10 μm. **(D-E)** Dorsal sections of the SEZ, PENP and protocerebral bridge region from the same brain as in B. There are no obvious changes in ventral OGN survival and differentiation as measured by Tdc2 expression. Scale bar = 20 μm.
(TIF)

S6 Fig **(A)** Verification that each *tdc2>GFP* neuron in the brain and VNS is Tdc2+. The stack for panel A contains 30 optical sections at 1.0 μm. Scale bar = 20 μm. **(B)** The stack for panel B contains 34 optical sections at 1.0 μm. Scale bar = 20 μm. **(C-E)** Verification that each *tdc2-dVGlut-split>GFP* neuron is Tdc2+. The stack for panels C-E contains 56 optical sections at 0.5 μm. Scale bar = 20 μm. **(F)** Schematic showing the locations of Tdc+ clusters in C-E.
(TIF)

S7 Fig **(A)** The activity levels of controls and *tdc2>dVGlut-RNAi* males did not differ during the aggression assay as measured by pixels moved/second. **(B)** Total behavioral events (lunges, wing threats, inter-male courtship) per minute was calculated. The average number of behavioral events per minute exhibited by experimental males (*tdc2>tsh>Gal80>dVGlut-RNAi*) was slightly higher than controls (**p<0.01)
(TIF)

S8 Fig **(A)** The VNS of a *tdc2>mtd*:*HA* male, note the Tdc2+ cell bodies. **(B)** The addition of *tsh>Gal80* blocked the Gal4-mediated expression of mtd:HA in the majority of Tdc2+ VNS neurons (*tdc2/tsh>Gal80;dsRed)*. Axonal projections from brain Tdc2+ neurons are visualized in the VNS. **(C)** Significantly less Tdc2+ VNS neurons are detected in *tdc2/tsh>Gal80;dsRed* vs. *tdc2>dsRed* males. (Mann Whitney, P = 0.001). **(D)** The addition of *tsh>Gal80* does not alter brain *tdc2-gal4* reporter driven expression.
(TIF)

S9 Fig Neuron survival or distribution is not altered by the complete loss of dVGLUT in OGNs **(A-D)** Representative images of dorsal (A-B) and ventral (C-D) optical sections of the SEZ region from *tdc2-gal4;B3RT-dVGlut/dVGLUT*^*SS1*^*;UAS-B3 lexAop-His2B-mCherry UAS-His2A-GFP* males. OGNs are visualized by the mCherry reporter and white co-colocalization in the merged channel. Scale bar = 20 μm.
(TIF)

S10 Fig RSRT>stop>6xV5-VMAT is not expressed without Gal4-mediated excision of the stop cassette. **(A-A’)** In the presence of a Gal4 driver (*tdc2-Gal4-AD dVGlut-Gal4-DBD*) to drive R recombinase (*UAS-R*) expression, the stop cassette of RSRT>stop>6XV5-VMAT is excised and V5-VMAT (magenta) is expressed and visualized by anti-V5. dVGLUT (green) is visualized by mAb dVGLUT. **(B-B’)** Without the presence of a Gal4 driver, dVGLUT expression is apparent while expression from RSRT>stop>6XV5-VMAT is not detected by anti-V5. Scale bar = 30 μm.
(TIF)

S11 Fig **(A)** Higher magnification of the SEZ region showing V5-VMAT expression in OGNs after excision by *tdc2-dVGlut-gal4* driven R recombinase. The brain is labeled with anti-V5 (magenta) and mAb dVGLUT (green). Scale bar = 15 μm**. (B-B”)** Higher magnification of the SEZ region of the region in the dashed box in panel B. Arrowheads indicate puncta with dVGLUT and V5-VMAT colocalization. Arrows indicate puncta with only V5-VMAT (arrows). **(C)** Schematic indicating the location of the SEZ region. **(D)** SEZ region of a representative brain with a synaptic marker incorporated (*UAS-synaptotagmin;HA*, *tdc2-dVGlut-gal4/UAS-R RSRT-STOP-RSRT-6XV5-vMAT)*. The brain is labeled with anti-HA (blue), anti-V5 (magenta), and mAb dVGLUT (green). Scale bar = 20 μm. **(E)** Higher magnification of the SEZ region in D. Scale bar = 10 μm. **(F-H**) Regions of interest from E showing puncta with dVGLUT, V5-VMAT and Syt:HA. The stack for panel B contains two optical sections at 0.45 μm. Six optical sections at 0.45 μm were stacked in panels E-H.
(TIF)

S12 Fig OGNs include the three OA-FruM^+^ neurons. **(A-C)** Brains from *tdc2-dVGlut-split-gal4/UAS>stop>CD8*:*GFP;fru-flp* males demonstrate OA-FruM^+^ neurons are also dVGlut+. **(D)** No OGNs in the VNS are FruM^+^ although as expected the OGN-FruM^+^ neurons project into the VNS. Scale bar = 20 μm. **(E-G)** OGN-FruM+ neurons (arrow) were also identified in *dVGlut-gal4/UAS>stop>CD8*:*GFP;fru-flp* male brains labeled with anti-Tdc2 (magenta). Scale bar = 20 μm.
(TIF)

S1 Table Identified OGNs based on OA neuron nomenclature. (TIF)

S2 Table Cloning components used for the construction of the 20XUAS-His2A-GFP and 13XLexAop2-His2B-mCherry lines. (TIF)

S1 Data (TIF)
