# Supplementary figures and images for: Octopamine neuron dependent aggression requires dVGLUT from dual-transmitting neurons
Source: PLoS Genet. 2020 Feb 25;16(2):e1008609. doi: 10.1371/journal.pgen.1008609 (PMC7059954; doi:10.1371/journal.pgen.1008609)

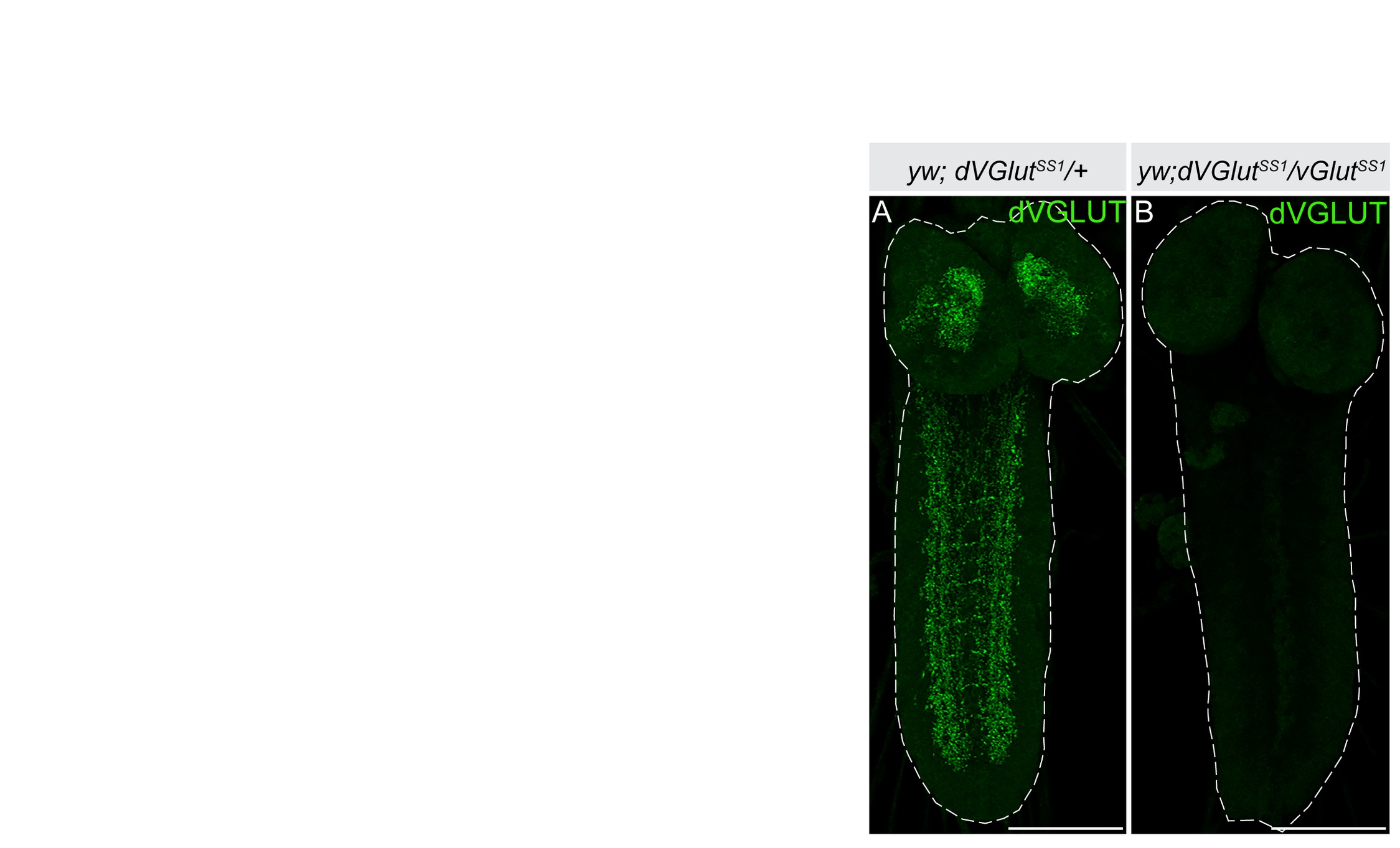

Supplement: S1 Fig — (A) dVGLUT expression detected by mAb dVGLUT in a heterozygous yw, dVGlutSS1/+ late stage embryo. (B) dVGLUT expression is not detectable by mAb dVGLUT in a homozygous yw, dVGlutSS1/ dVGlutSS1 late stage embryo. (TIF) [file pgen.1008609.s001.tif]

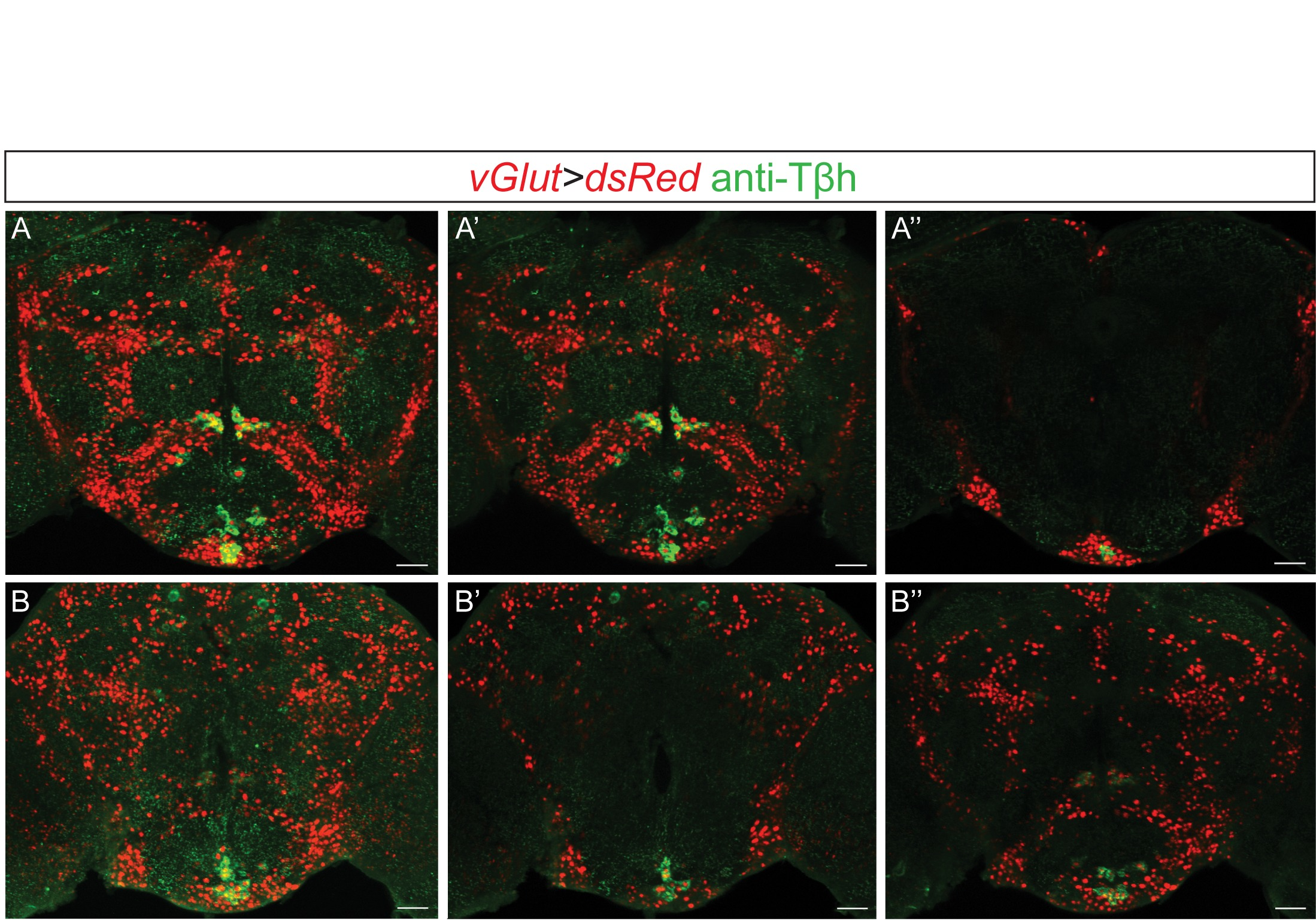

Supplement: S2 Fig — (A-B) Although the Tβh shows weaker immunoreactivity than the anti-Tdc2 antibody, Tβh is mainly detected in dVGlut>dsRed neurons at dorsal and ventral positions (A’, A”, B’ and B”). Scale bar = 20 μm. (TIF) [file pgen.1008609.s002.tif]

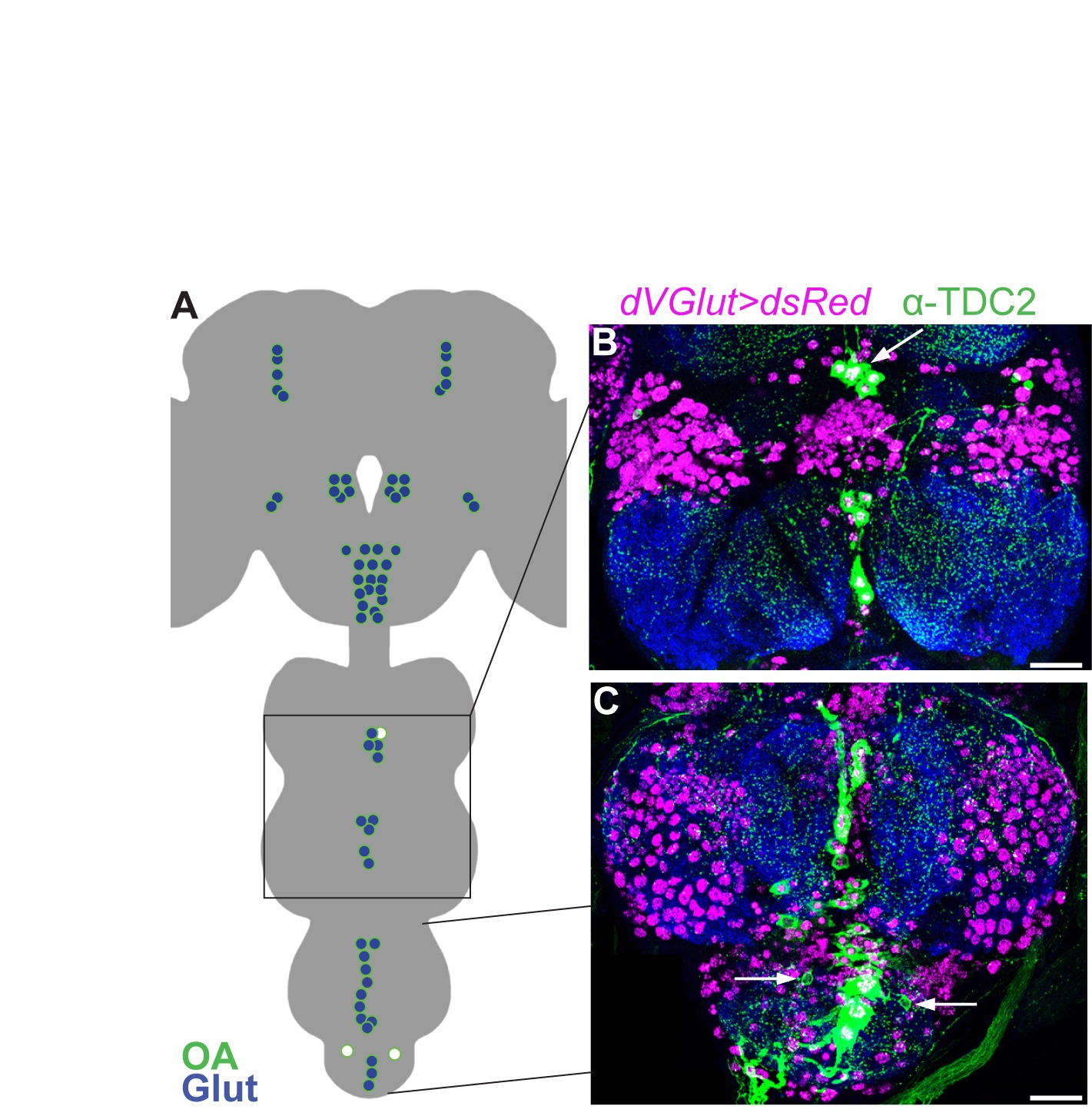

Supplement: S3 Fig — (A-A’) Schematic showing the regions (boxes) of the VNS imaged in panels B and C. (B-C) A male dVGlut>dsRed adult VNS labeled with anti-Tdc2. The majority of dVGLUT+ neurons within the thoracic VNS (B) and abdominal VNS (C) express Tdc2 with a few exceptions (arrows). Scale bar = 10 μm. (TIF) [file pgen.1008609.s003.tif]

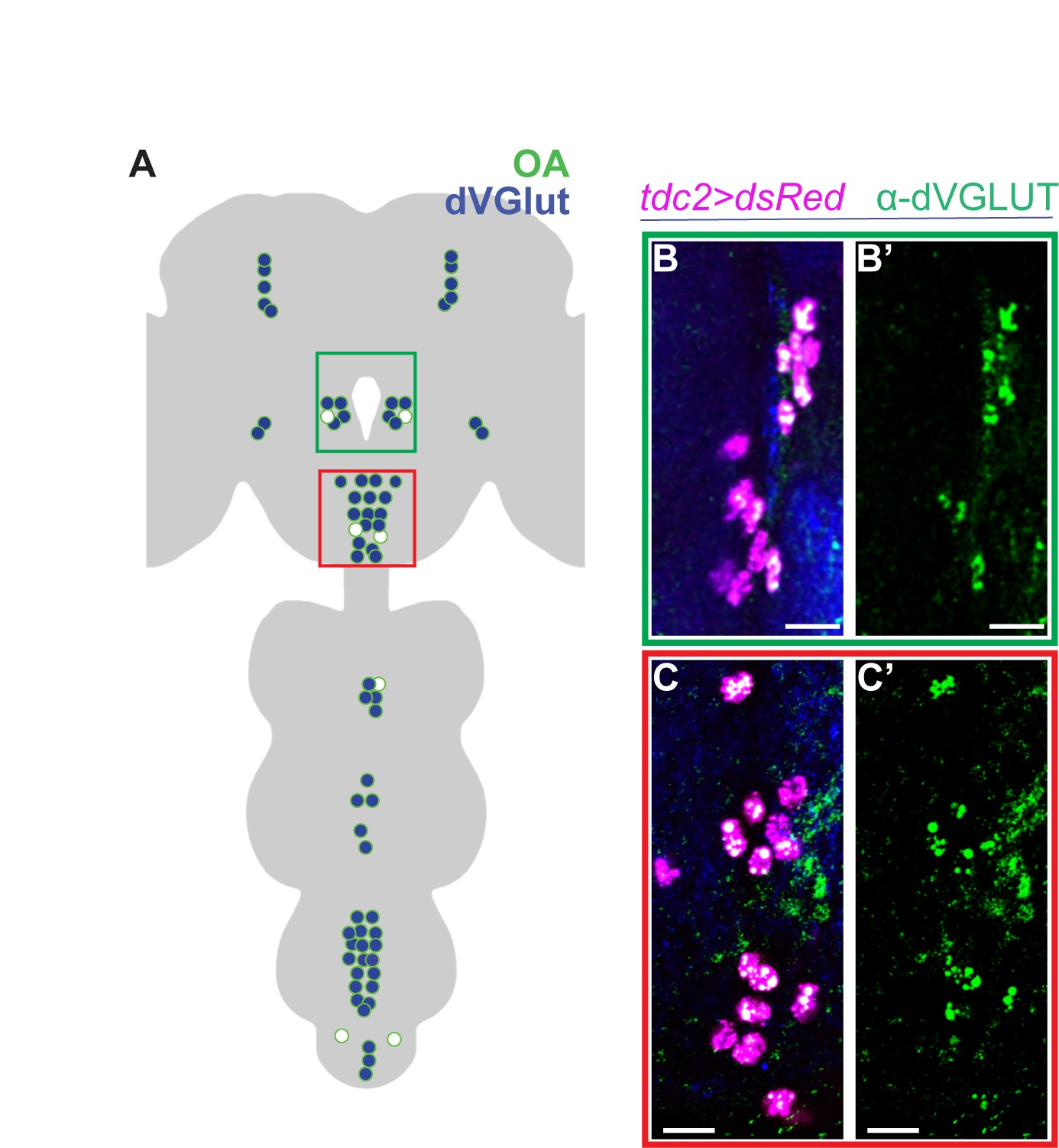

Supplement: S4 Fig — (A) Schematic showing the regions imaged in panels B and C (colored boxes). (B-C) The majority of OA neurons within the PENP (B) and SEZ (C) regions co-express dVGLUT as visualized in a male tdc2>dsRed adult brain labeled with anti-dVGLUT. Scale bar = 10 μm. (TIF) [file pgen.1008609.s004.tif]

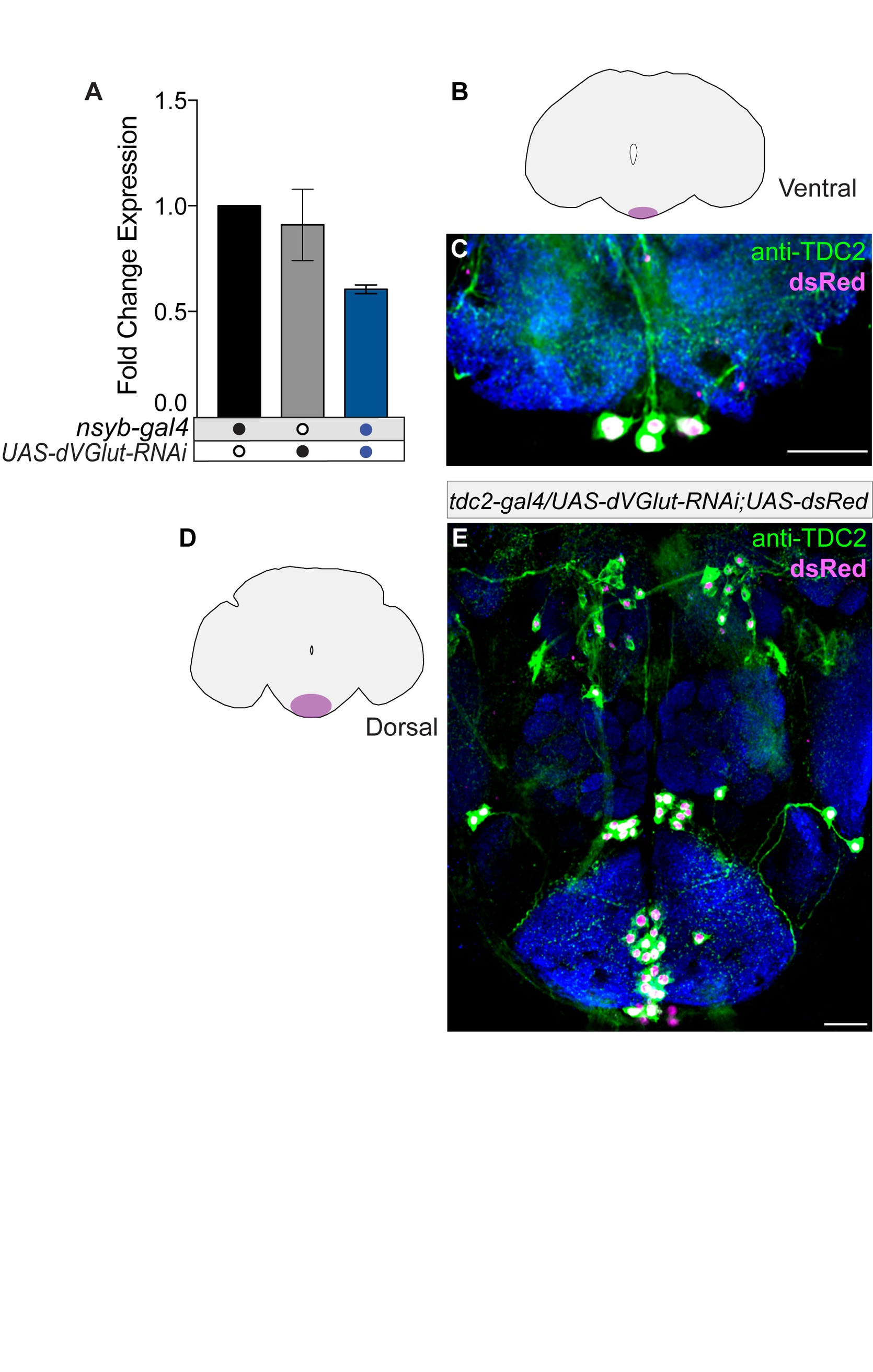

Supplement: S5 Fig — (A) dVGlut transcript levels were decreased in n-syb-gal4>dVGLUT-RNAi males as compared to the n-syb-gal4 control (n = 3; p<0.01). (B-C) Representative images of ventral sections of the SEZ from a tdc2-gal4>dVGLUT-RNAi;UAS-dsRed male brain labeled with anti-Tdc2. OGN differentiation as measured by Tdc2 expression is not altered by a reduction of dVGLUT. Scale bar = 10 μm. (D-E) Dorsal sections of the SEZ, PENP and protocerebral bridge region from the same brain as in B. There are no obvious changes in ventral OGN survival and differentiation as measured by Tdc2 expression. Scale bar = 20 μm. (TIF) [file pgen.1008609.s005.tif]

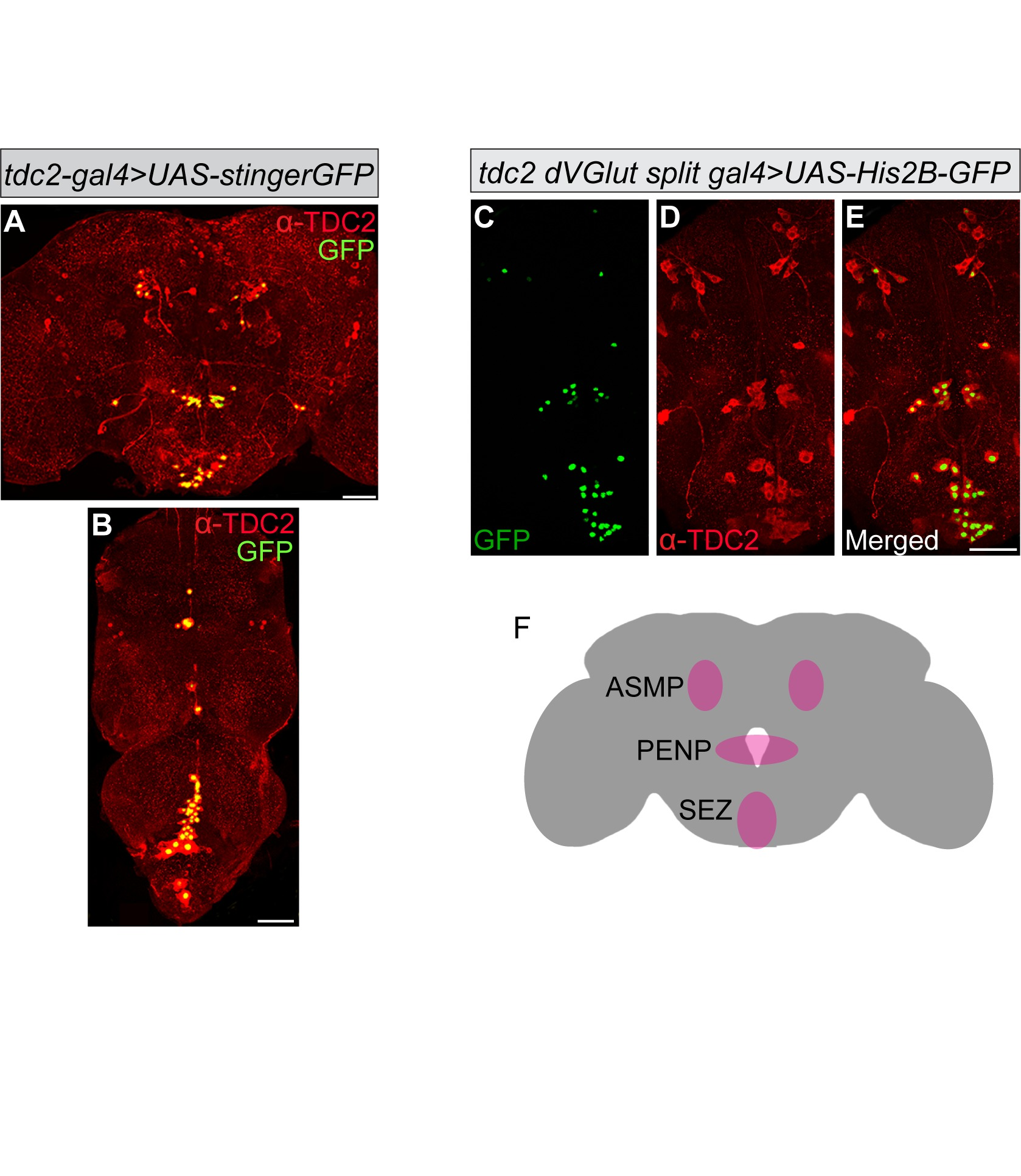

Supplement: S6 Fig — (A) Verification that each tdc2>GFP neuron in the brain and VNS is Tdc2+. The stack for panel A contains 30 optical sections at 1.0 μm. Scale bar = 20 μm. (B) The stack for panel B contains 34 optical sections at 1.0 μm. Scale bar = 20 μm. (C-E) Verification that each tdc2-dVGlut-split>GFP neuron is Tdc2+. The stack for panels C-E contains 56 optical sections at 0.5 μm. Scale bar = 20 μm. (F) Schematic showing the locations of Tdc+ clusters in C-E. (TIF) [file pgen.1008609.s006.tif]

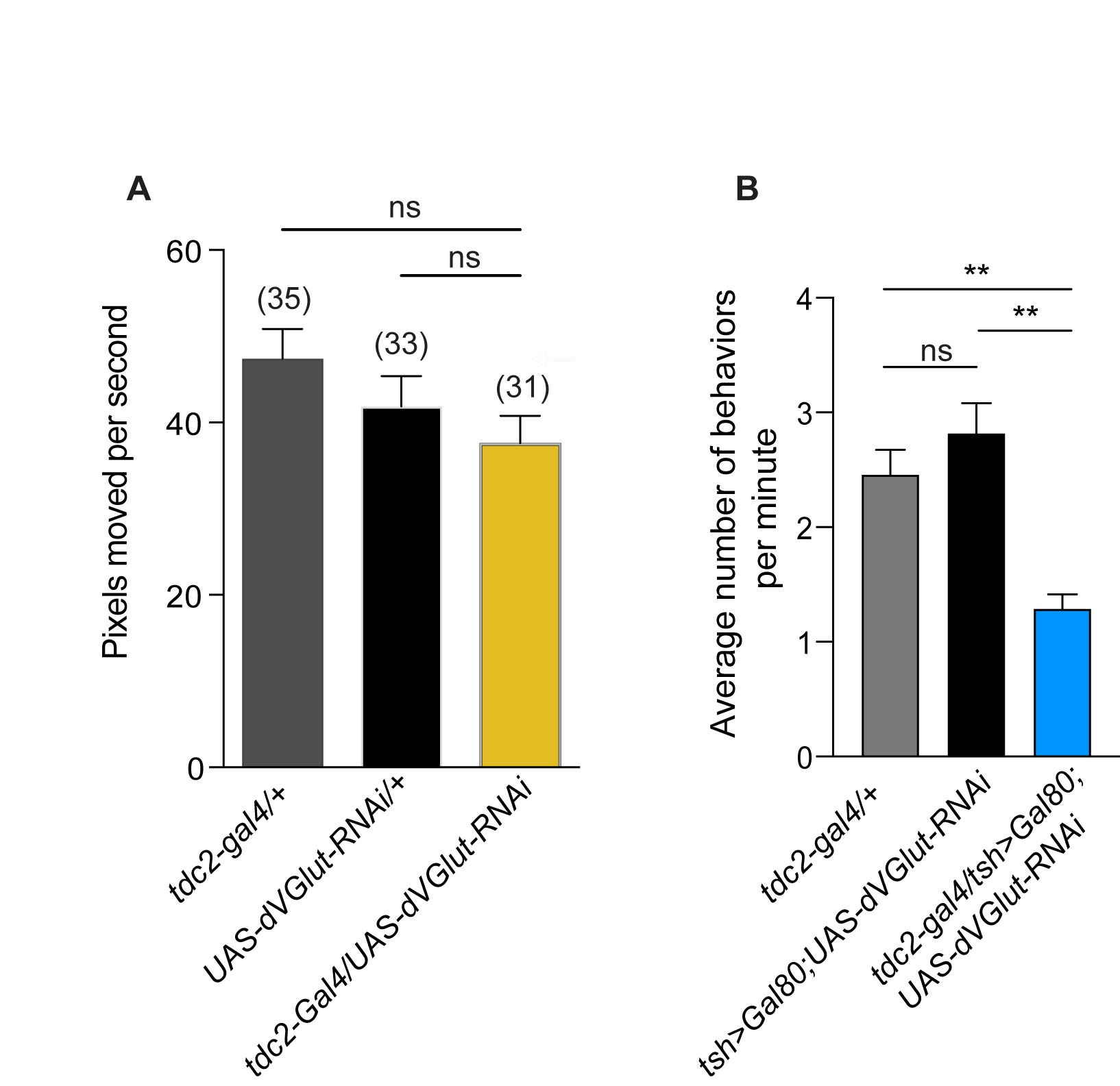

Supplement: S7 Fig — (A) The activity levels of controls and tdc2>dVGlut-RNAi males did not differ during the aggression assay as measured by pixels moved/second. (B) Total behavioral events (lunges, wing threats, inter-male courtship) per minute was calculated. The average number of behavioral events per minute exhibited by experimental males (tdc2>tsh>Gal80>dVGlut-RNAi) was slightly higher than controls (**p<0.01) (TIF) [file pgen.1008609.s007.tif]

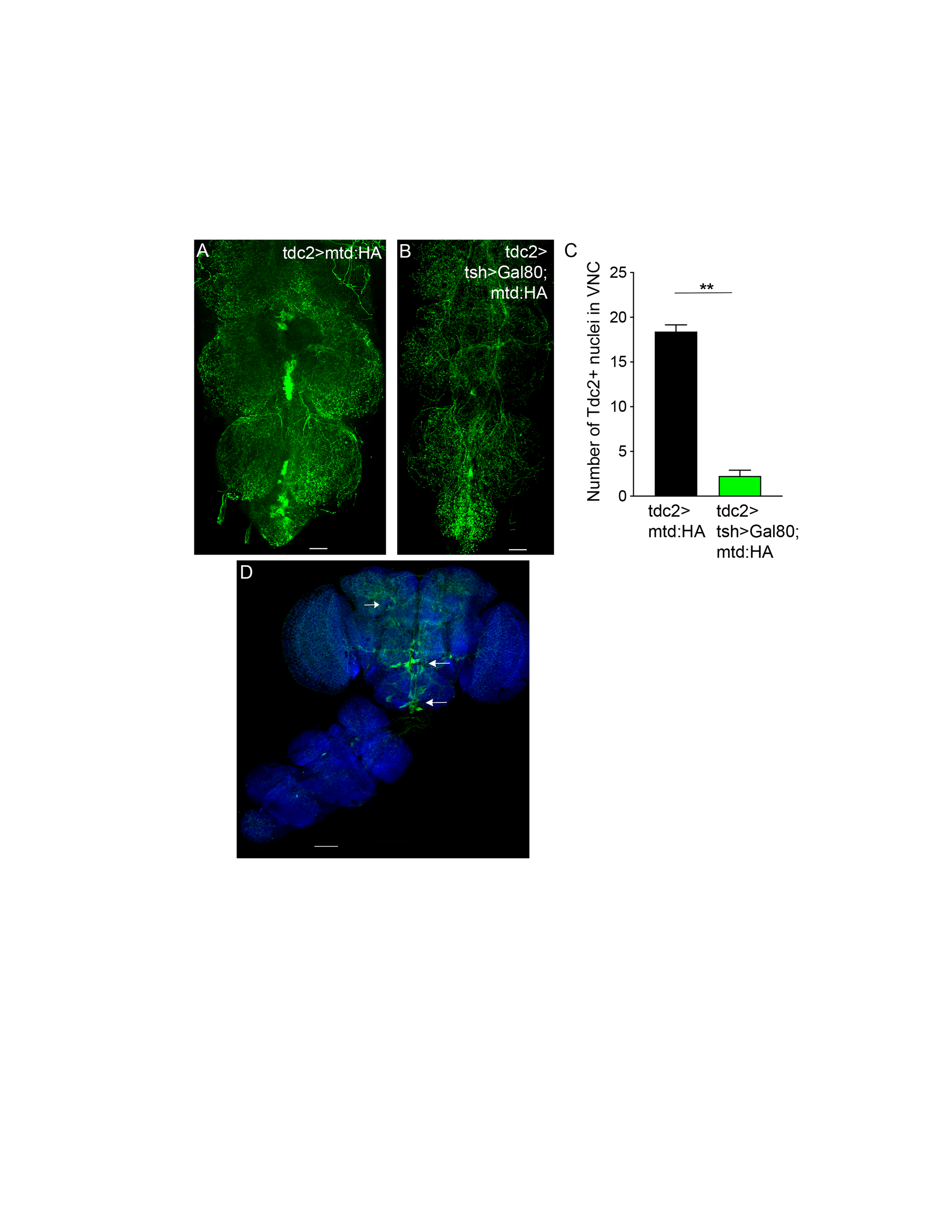

Supplement: S8 Fig — (A) The VNS of a tdc2>mtd:HA male, note the Tdc2+ cell bodies. (B) The addition of tsh>Gal80 blocked the Gal4-mediated expression of mtd:HA in the majority of Tdc2+ VNS neurons (tdc2/tsh>Gal80;dsRed). Axonal projections from brain Tdc2+ neurons are visualized in the VNS. (C) Significantly less Tdc2+ VNS neurons are detected in tdc2/tsh>Gal80;dsRed vs. tdc2>dsRed males. (Mann Whitney, P = 0.001). (D) The addition of tsh>Gal80 does not alter brain tdc2-gal4 reporter driven expression. (TIF) [file pgen.1008609.s008.tif]

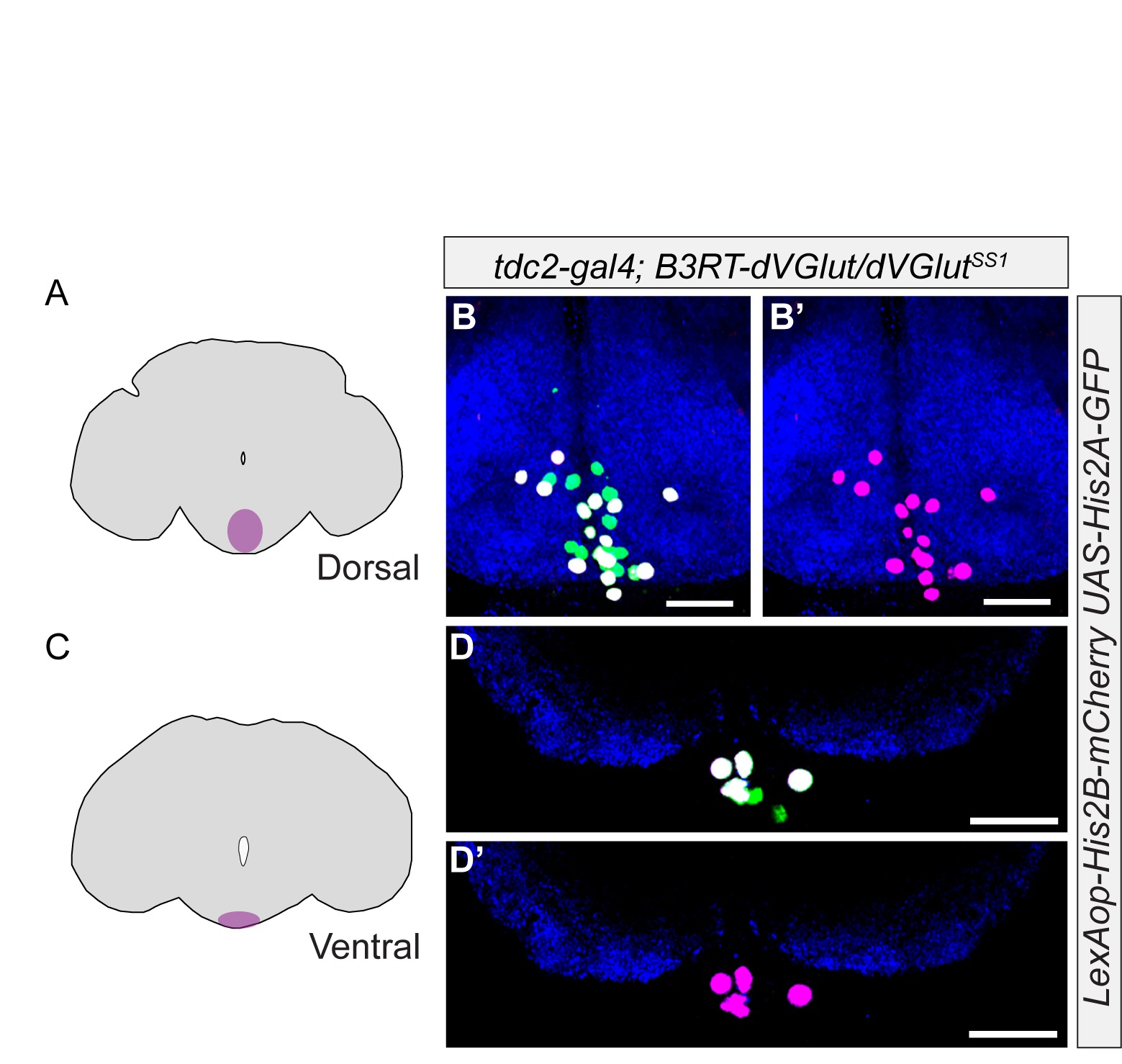

Supplement: S9 Fig — Neuron survival or distribution is not altered by the complete loss of dVGLUT in OGNs (A-D) Representative images of dorsal (A-B) and ventral (C-D) optical sections of the SEZ region from tdc2-gal4;B3RT-dVGlut/dVGLUTSS1;UAS-B3 lexAop-His2B-mCherry UAS-His2A-GFP males. OGNs are visualized by the mCherry reporter and white co-colocalization in the merged channel. Scale bar = 20 μm. (TIF) [file pgen.1008609.s009.tif]

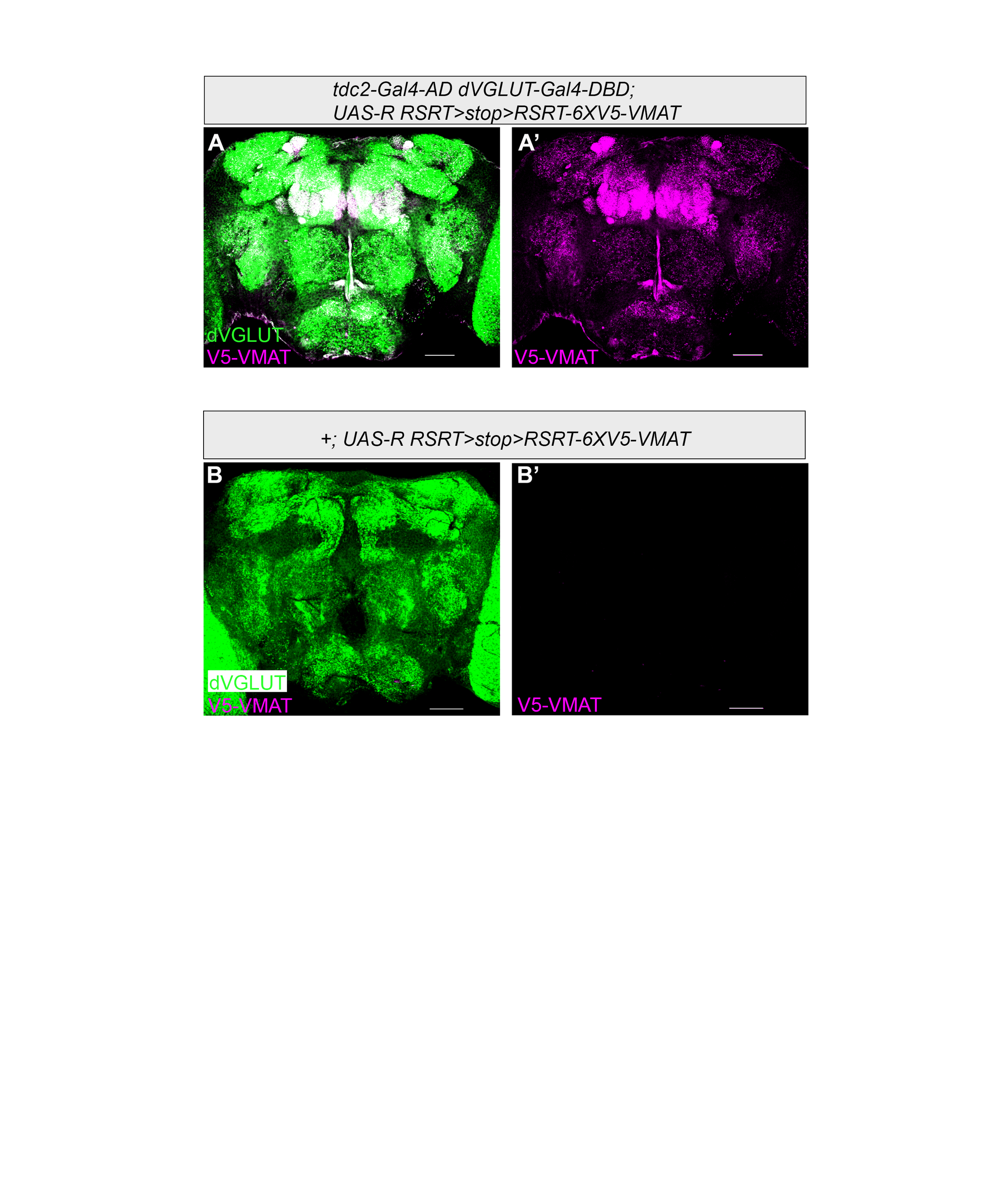

Supplement: S10 Fig — (A-A’) In the presence of a Gal4 driver (tdc2-Gal4-AD dVGlut-Gal4-DBD) to drive R recombinase (UAS-R) expression, the stop cassette of RSRT>stop>6XV5-VMAT is excised and V5-VMAT (magenta) is expressed and visualized by anti-V5. dVGLUT (green) is visualized by mAb dVGLUT. (B-B’) Without the presence of a Gal4 driver, dVGLUT expression is apparent while expression from RSRT>stop>6XV5-VMAT is not detected by anti-V5. Scale bar = 30 μm. (TIF) [file pgen.1008609.s010.tif]

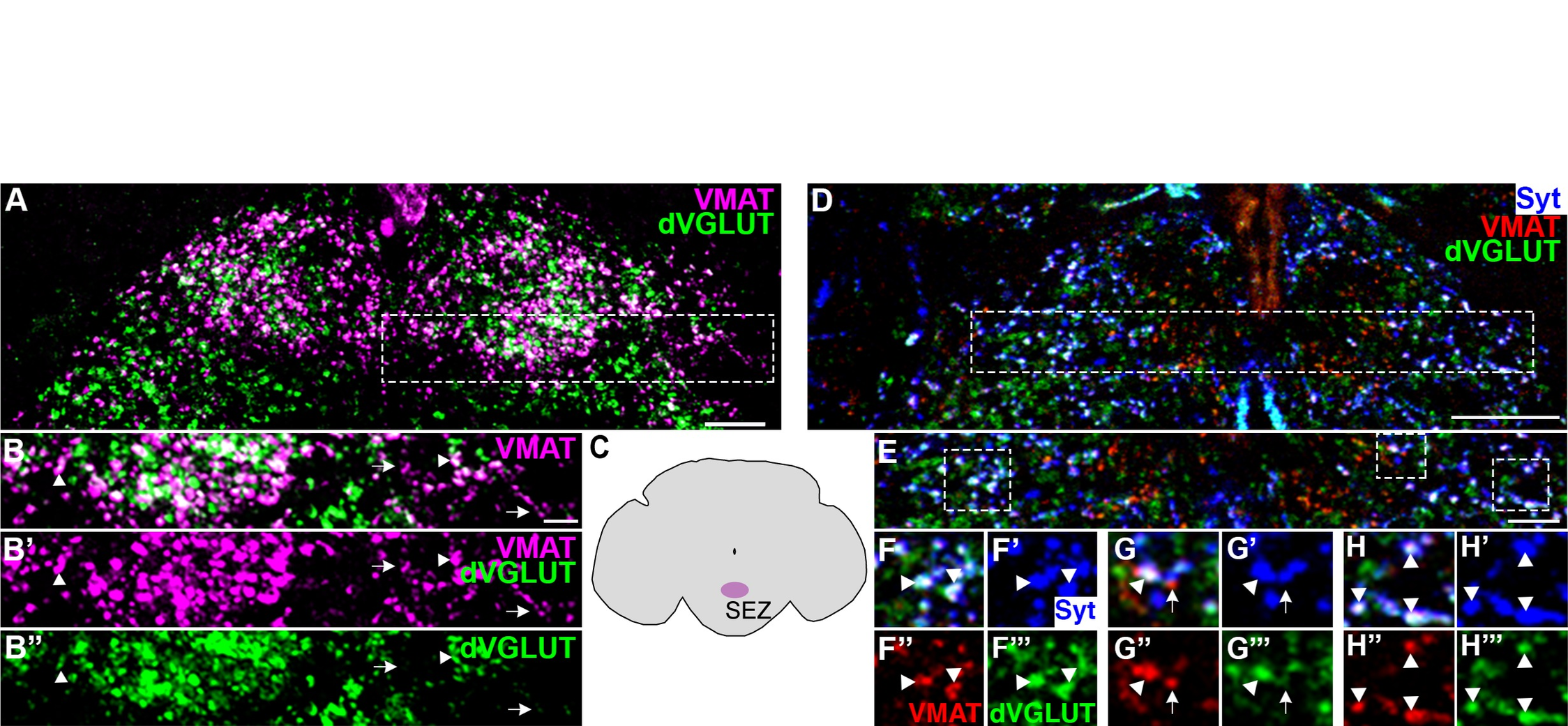

Supplement: S11 Fig — (A) Higher magnification of the SEZ region showing V5-VMAT expression in OGNs after excision by tdc2-dVGlut-gal4 driven R recombinase. The brain is labeled with anti-V5 (magenta) and mAb dVGLUT (green). Scale bar = 15 μm. (B-B”) Higher magnification of the SEZ region of the region in the dashed box in panel B. Arrowheads indicate puncta with dVGLUT and V5-VMAT colocalization. Arrows indicate puncta with only V5-VMAT (arrows). (C) Schematic indicating the location of the SEZ region. (D) SEZ region of a representative brain with a synaptic marker incorporated (UAS-synaptotagmin;HA, tdc2-dVGlut-gal4/UAS-R RSRT-STOP-RSRT-6XV5-vMAT). The brain is labeled with anti-HA (blue), anti-V5 (magenta), and mAb dVGLUT (green). Scale bar = 20 μm. (E) Higher magnification of the SEZ region in D. Scale bar = 10 μm. (F-H) Regions of interest from E showing puncta with dVGLUT, V5-VMAT and Syt:HA. The stack for panel B contains two optical sections at 0.45 μm. Six optical sections at 0.45 μm were stacked in panels E-H. (TIF) [file pgen.1008609.s011.tif]

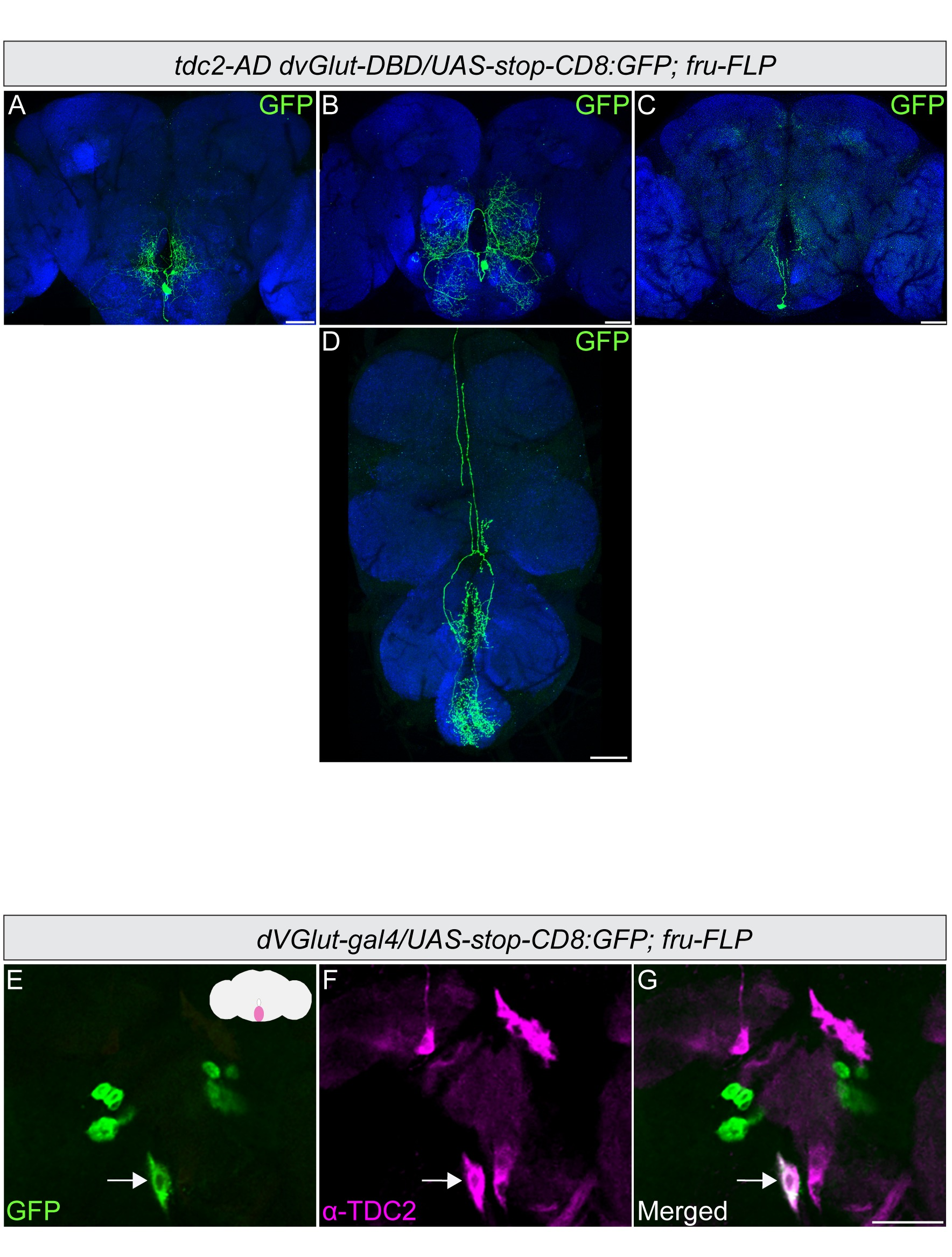

Supplement: S12 Fig — (A-C) Brains from tdc2-dVGlut-split-gal4/UAS>stop>CD8:GFP;fru-flp males demonstrate OA-FruM+ neurons are also dVGlut+. (D) No OGNs in the VNS are FruM+ although as expected the OGN-FruM+ neurons project into the VNS. Scale bar = 20 μm. (E-G) OGN-FruM+ neurons (arrow) were also identified in dVGlut-gal4/UAS>stop>CD8:GFP;fru-flp male brains labeled with anti-Tdc2 (magenta). Scale bar = 20 μm. (TIF) [file pgen.1008609.s012.tif]

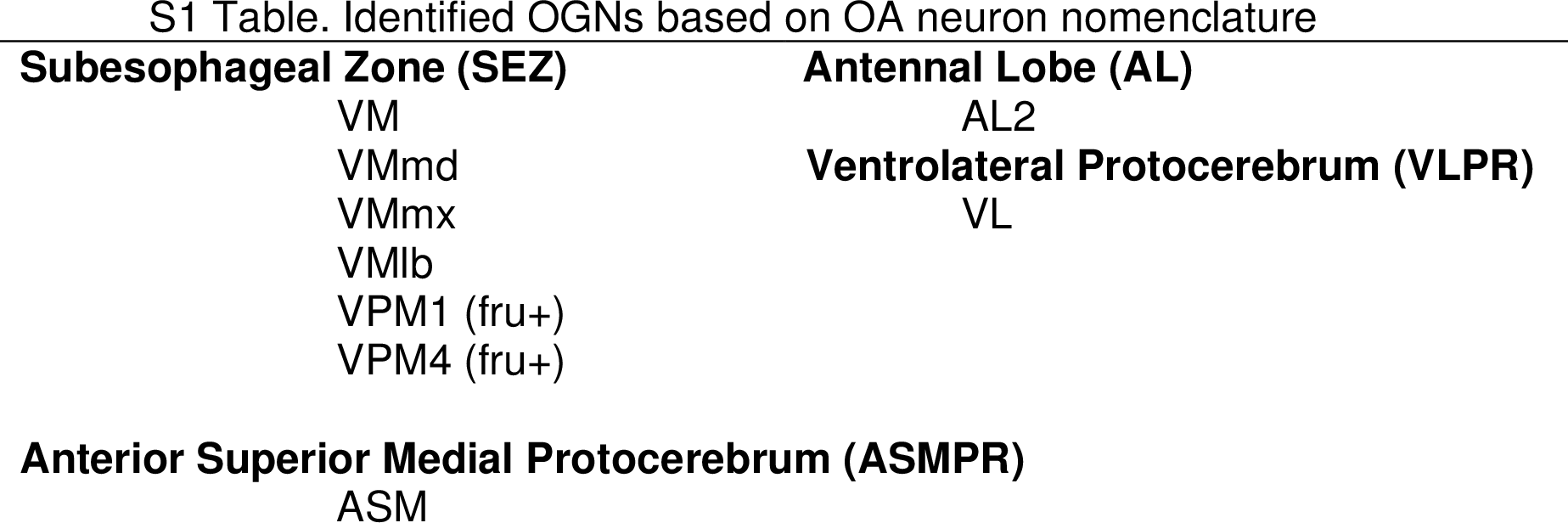

Supplement: S1 Table — (TIF) [file pgen.1008609.s013.tif]

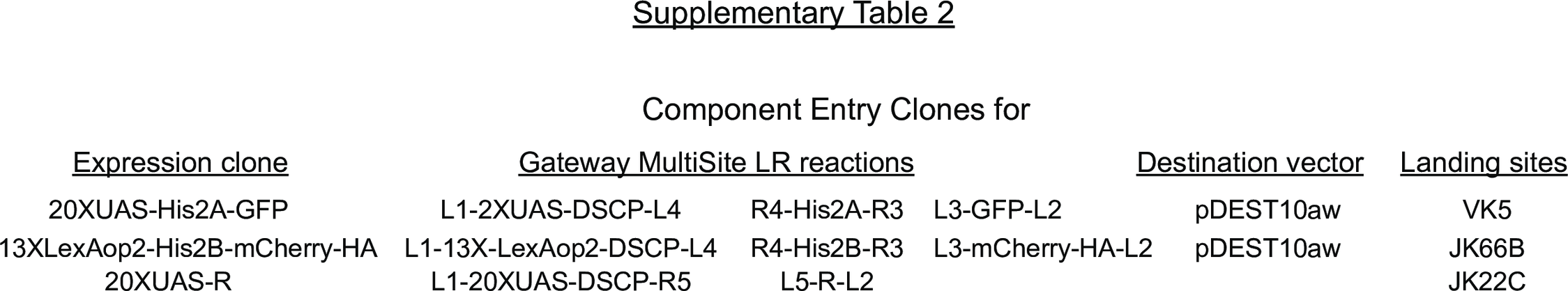

Supplement: S2 Table — (TIF) [file pgen.1008609.s014.tif]

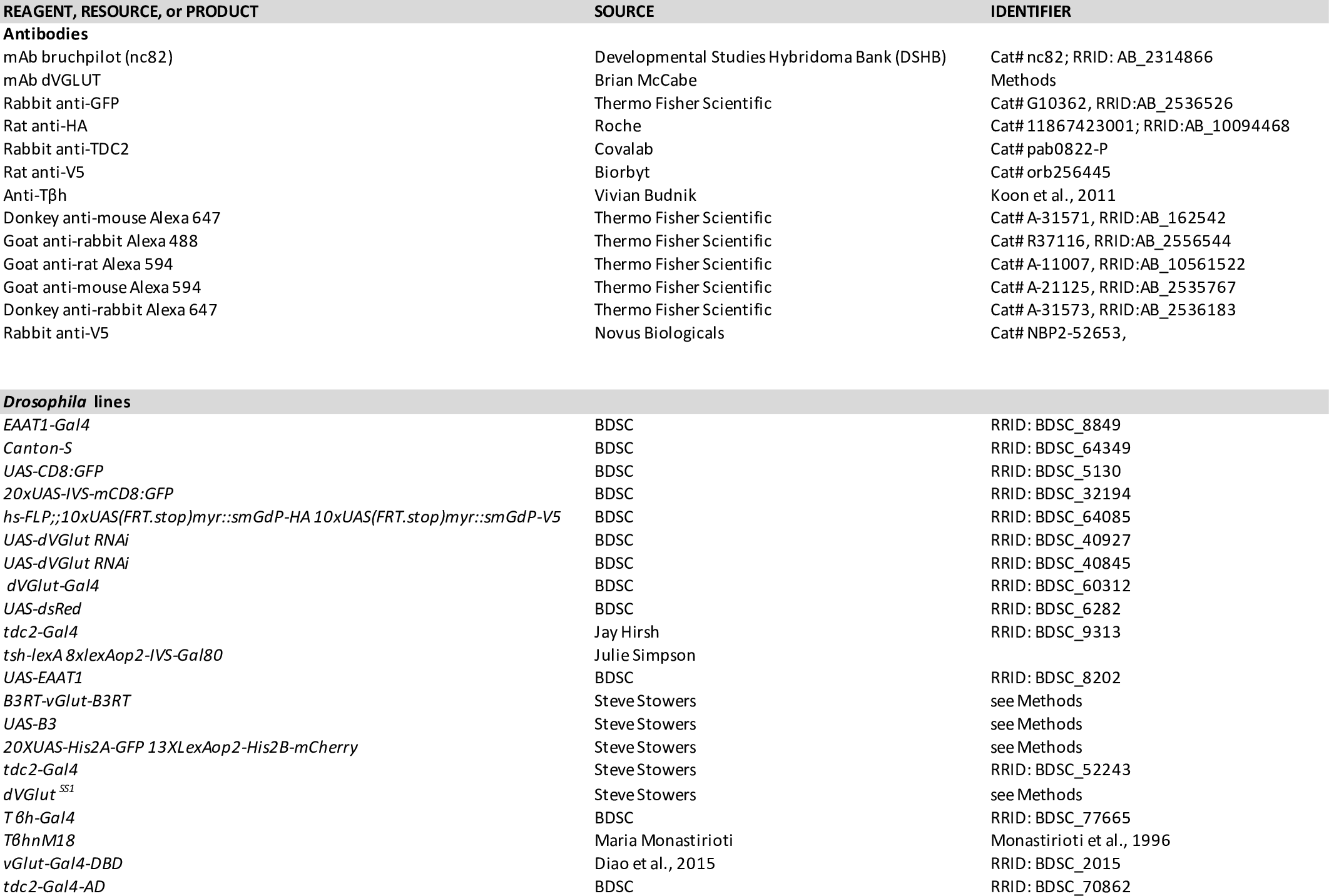

Supplement: S1 Data — (TIF) [file pgen.1008609.s015.tif]
